# Molecular Deformation Is a Key Factor in Screening Aggregation Inhibitor for Intrinsically Disordered Protein Tau

**DOI:** 10.1021/acscentsci.3c01196

**Published:** 2024-03-05

**Authors:** Keke Chai, Jian Yang, Ying Tu, Junjie Wu, Kang Fang, Shuo Shi, Tianming Yao

**Affiliations:** † School of Chemical Science and Engineering, Shanghai Key Laboratory of Chemical Assessment and Sustainability, Tongji University, Shanghai 200092, China; ‡ School of Medicine, Shanghai University, Shanghai 200444, China

## Abstract

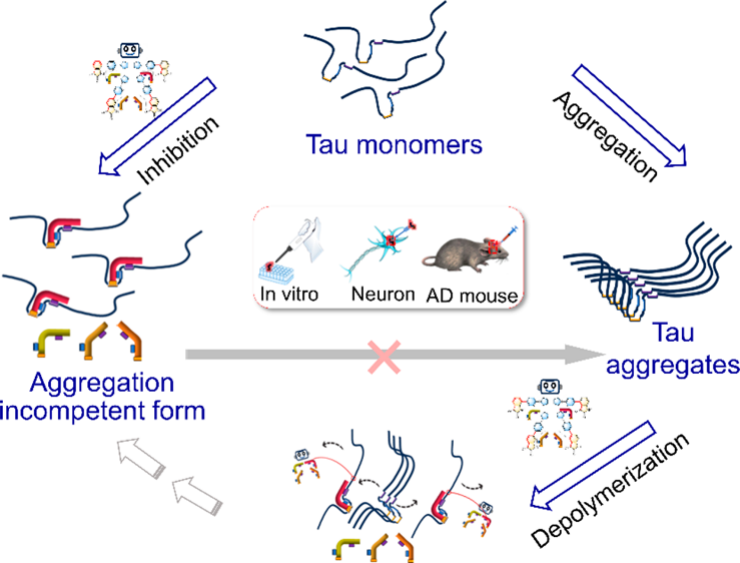

Direct inhibitor of tau aggregation has been extensively studied as potential therapeutic agents for Alzheimer’s disease. However, the natively unfolded structure of tau complicates the structure-based ligand design, and the relatively large surface areas that mediate tau–tau interactions in aggregation limit the potential for identifying high-affinity ligand binding sites. Herein, a group of isatin-pyrrolidinylpyridine derivative isomers (IPP1–IPP4) were designed and synthesized. They are like different forms of molecular “transformers”. These isatin isomers exhibit different inhibitory effects on tau self-aggregation or even possess a depolymerizing effect. Our results revealed for the first time that the direct inhibitor of tau protein aggregation is not only determined by the previously reported conjugated structure, substituent, hydrogen bond donor, etc. but also depends more importantly on the molecular shape. In combination with molecular docking and molecular dynamics simulations, a new inhibition mechanism was proposed: like a “molecular clip”, IPP1 could noncovalently bind and fix a tau polypeptide chain at a multipoint to prevent the transition from the “natively unfolded conformation” to the “aggregation competent conformation” before nucleation. At the cellular and animal levels, the effectiveness of the inhibitor of the IPP1 has been confirmed, providing an innovative design strategy as well as a lead compound for Alzheimer’s disease drug development.

## Abstract 2

We propose that molecular deformation is a key factor in the screening aggregation inhibitor for intrinsic disordered protein tau. We designed and synthesized four isomers with different shapes by a modular combination of isatin and pyrrolidinylpyridine and verified that they have different binding abilities to tau and inhibitory activities against tau aggregation. Our results will provide a new direction for developing a tau aggregation inhibitor.

## Introduction

Tau protein is a microtubule-associated protein that is mainly expressed in central and peripheral nerve systems. Under physiological conditions, tau binds to tubulin and stabilizes microtubules, thus playing a critical role in neuron morphology, axon development, and navigation.^1^ In its native conformation, tau is largely unfolded, highly disordered, and stable.^2^ However, in the numerous neuropathologies known as tauopathies, tau is aggregated into amyloid fibrils, which in turn may produce severe pathologies known as protein misfolding diseases. The most-prevalent tauopathy is Alzheimer’s disease (AD), which severely damages the cognitive function of the elderly and threatens their health.^3^

Various laboratories are studying the possibility of the direct inhibition of tau aggregation utilizing small molecules that could be developed into AD drugs. Having the advantage of disease specificity, hundreds of seemingly unrelated small molecular inhibitors of tau aggregation have been disclosed.^4,5^ The compounds can be classified according to their chemical structures, including but not limited to polyphenols,^6^ anthraquinones,^7^ rhodanines,^8^ phenylthiazolyl-hydrazide,^9^ phenylamines,^10^ aminothienopyridazines,^11^ phenothiazines,^12^ porphyrins,^6^ and cyanines.^13^ Each chemotype differs in conjugated structure, substituent, hydrogen bond donor, hydrophobicity, molecular size, and weight. These small molecules inhibit tau aggregation *in vitro* at micromolar or even submicromolar concentrations, and some of them are in clinical trials. Although finding small molecules to directly inhibit tau aggregation is feasible, screening approaches are expensive in terms of effort, time, and cost.

The first step of drug development is a rational structure-guided strategy to identify and optimize small molecular ligands. One of the major hurdles faced by the approach is the lack of distinguishable “binding pockets” on the tau monomer, according to the “lock and key” mechanism for well-folded proteins in conventional drug discovery. The natively unfolded structure of the tau target complicates the structure-based ligand design, and the relatively large surface areas that mediate tau–tau interactions in aggregation limit the potential for identifying high-affinity ligand binding sites. Although previous work has shown that tau’s sequence segments ^275^VQIINK^280^ and ^306^VQIVYK^311^ drive its aggregation, but inhibitors based on the segments only partially inhibit full-length tau aggregation and are ineffective at inhibiting seeding by full-length fibrils.^14^

The current literature studies on tau aggregation inhibitors mainly focus on the thermodynamic docking relationship between inhibitors (ligands) and protein receptors.^15^ However, statistics reveal that there is no intrinsic relationship between the binding constant (*K*_b_) and the inhibition efficacy (IC_50_); that is, the stronger binding force does not necessarily result in a higher inhibition potency.^16^ Meanwhile, it has been proposed that even transient interactions could depress entry into aggregation pathways by altering the rate at which natively unfolded polypeptides adopt aggregation competent conformations.^17^ We have suggested a new strategy in light of the “new view” of the kinetic mechanism for reducing tau aggregation. We have proved that glucose gallate can inhibit tau aggregation by reducing the flexibility of the peptide chain as a “molecular scaffold”, thereby enhancing the energy barrier in the dynamic pathway of interconversion between aggregation competent and incompetent conformations.^18^ Schafer et al. suggested that flat, highly polarizable ligands inhibited tau aggregation by interacting with folded species in the aggregation pathway, driving their assembly into soluble oligomers along an off-aggregation pathway. Using structure–activity relationship analysis, they identified polarizability as a common descriptor of inhibitor potency. However, their speculation was based only on noncovalent inhibition mediated in part by the π–π stacking interaction of highly polarizable phenylalanine and tyrosine residues among the tau amino acid sequence.^16^

Overall, the previous screening of tau aggregation inhibitors originated from a distinct molecular skeleton of natural products or synthetic compounds, followed by changes in conjugated systems or substituents around it to increase the candidate’s molecular diversity. Recently, we have questioned how inhibitory efficacy or potency is achieved across scaffold classes of molecular ligands. Here we would like to innovatively propose a new hypothesis; that is, the spacing fit principle in “complementarity base-pairing” in the DNA double helix may be equally fundamental to the close recognition between the drug ligand and targeted protein, especially for intrinsically disordered proteins such as tau. We will focus on the molecular shape determined by the position and orientation of its constituent fragments with different characteristics, i.e., positive/negative charge, hydrogen bond donor/acceptor, and a conjugated system. Each of the constituent fragments can be regarded as an element for the origination of supramolecular interaction. If each of the elements within the molecule ligand fits neatly to those within the peptide reception domain to maintain the correct spacing, then there exist maximum supramolecular interactions, namely, van der Waals forces. Consequently, there should be the strongest noncovalent binding between the molecular ligand and protein receptor. In general, van der Waals forces can be divided into the electrostatic force, hydrogen bonding, π–π stacking, and hydrophilic/hydrophobic interaction. Therefore, the potency of the direct inhibitor of tau protein aggregation is determined not only by the characteristics of constitution fragments such as the conjugated system, substituent group, hydrogen bond donor, etc. as previously reported but also depends more importantly on their spatial arrangement, that is, the molecular shape determined by the position and orientation of each constitution fragments.

To confirm the above hypothesis and verify the decisive role of molecular shape in inhibiting tau aggregation, we herein designed and synthesized a group of isatin-pyrrolidinylpyridine derivative isomers (IPP1–IPP4). Evidence has rendered isatin and pyrrolidinylpyridine as a promising class with diverse bioactivities including tau binding activity.^19,20^ Coupling the two as building blocks, our isatin-pyrrolidinylpyridine isomers have the same molecular weight and the same functional group but have different shapes like different forms of molecular “transformers”. It was found that these isatin-pyrrolidinylpyridine derivatives have different inhibitory effects on tau aggregation or even have a depolymerizing effect. By the molecular transformers, we demonstrated for the first time that the direct inhibitor of tau protein aggregation is not only determined by its molecular structural constitution but also more importantly depends on the molecular shape as well as the orientation and position between molecular functional fragments. In combination with molecular docking simulation, a new inhibition mechanism was proposed: like a “molecular clip”, the isatin-pyrrolidinylpyridine isomer (IPP1) could noncovalently bind and fix the tau polypeptide chain at a multipoint to prevent the transition from “natively unfolded conformation” to “aggregation competent conformation” before nucleation while the other isomers (IPP2–IPP4) could not, emphasizing that the orientation of the molecular functional fragments is pivotal to forming a “molecular clip” and determines the effectiveness of direct inhibitors. At the in vitro, cellular, and animal levels, our pilot work has confirmed the effectiveness of IPP1 as a direct inhibitor of tau aggregation, providing a lead compound and even an innovative design strategy of drug development targeting the intrinsically disordered peptide chain in protein misfolding diseases, such as AD.

**Scheme 1:**
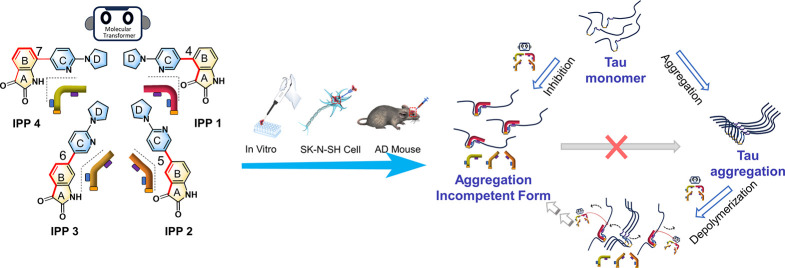
Screening the Tau Aggregation Inhibitor by Constructing Molecular Transformers Based on Isatin-pyrrolidinylpyridine Isomers

## Results and Discussion

### Design, Synthesis, and Structure of Isatin-pyrrolidinylpyridine Compounds

**Figure 1:**
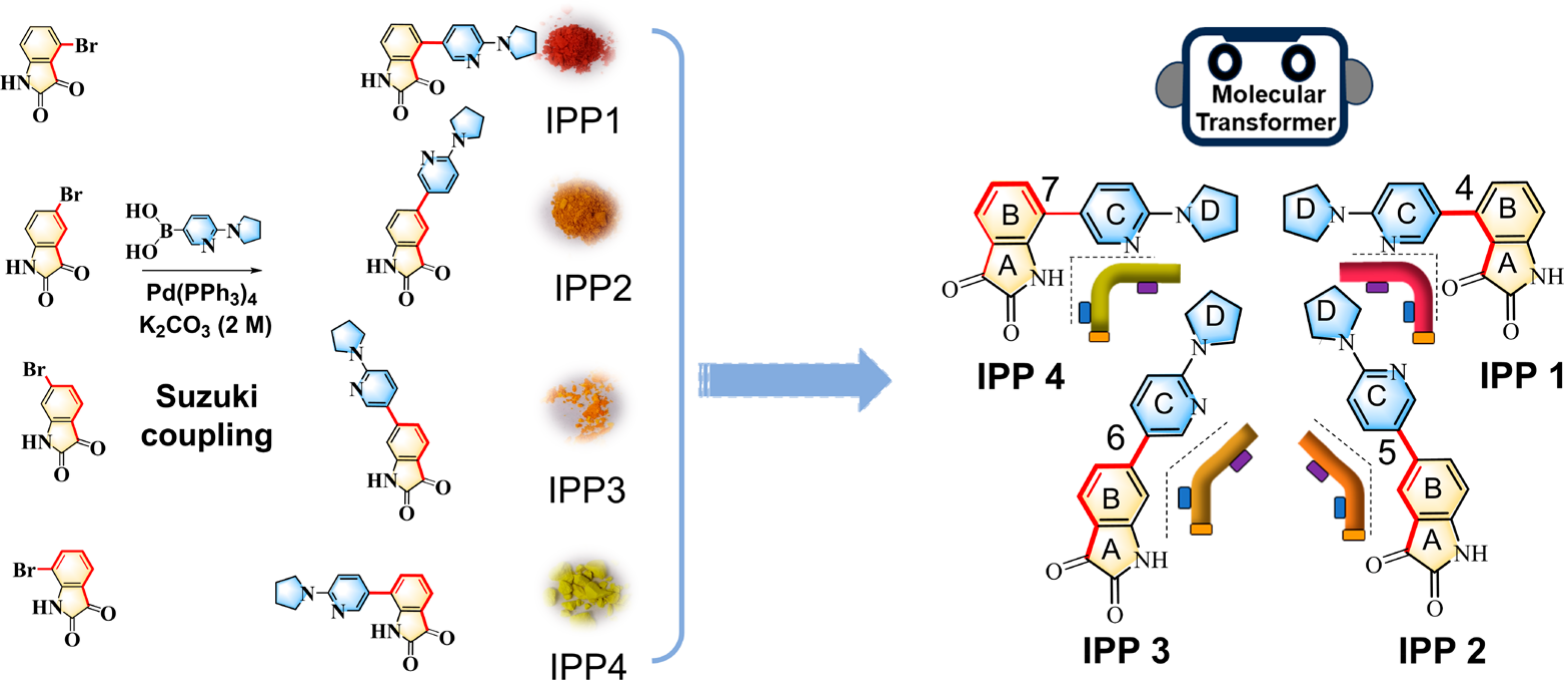
Synthesis of isatin-pyrrolidinylpyridine derivatives.

The isatin-pyrrolidinylpyridine compounds 4-(6-(pyrrolidin-1-yl)pyridin-3-yl)indoline-2,3-dione (IPP1), 5-(6-(pyrrolidin-1-yl)pyridin-3-yl)indoline-2,3-dione (IPP2), 6-(6-(pyrrolidin-1-yl)pyridin-3-yl)indoline-2,3-dione (IPP3), and 7-(6-(pyrrolidin-1-yl)pyridin-3-yl)indoline-2,3-dione (IPP4) were designed and synthesized by coupling two moieties, pyrrolidinylpyridine and isatin (Figure 1), along the Suzuki reaction according to the previously reported method with slight modifications.^21^ Detailed procedures and characterization data are given in the Supporting Information (Figures S1–12). These isomers have the same molecular weight and the same functional group but have different shapes such as different forms of molecular transformers.

The compounds consist of two fragments: isatin and pyrrolidinylpyridine. These two moieties were selected for us to design inhibitors targeting tau aggregates based on the following considerations: isatin (indole-2,3-dione) is an endogenous compound found in many organisms and has a wide range of biological activities.^22^ The isatin-containing derivative is also an effective inhibitor of cyclin-dependent kinases and glycogen synthase kinase.^23^ Nitrogen containing heterocycles are ubiquitous components of biologically active small molecules, which are particularly important for pharmaceutical applications. On the other hand, pyrazole and pyridine displayed good affinity toward tau aggregates and could be used as fluorescent probes for the detection of tau aggregates.^24,25^ In addition, nitrogen-containing heterocycles containing pyridine substituents exhibit good water solubility and lower toxicity.^26,27^ This evidence renders isatin and pyrrolidinylpyridine as promising blocks for constructing tau aggregation inhibitors.

In order to reveal the optimized molecular structures and unique electronic properties of the four isatin-pyrrolidinylpyridine compounds, we next conducted density functional theory calculations using Gauss 09 software at the B3LYP/6-31G level.^28^ By structural optimization, we find that twisted structure exists in all four isomers; however, high twisting exists in IPP1,^29^ which can be simply explained by the increase in steric hindrance.^30^ As for the distribution of electrons in molecules, it can be seen that only in IPP1 does the HOMO electron cloud extend to the carbonyl group in five-membered ring A of the isatin moiety while in the other isomers (IPP2–IPP4) the HOMO electron cloud is spread over the whole molecular skeleton, with a weak delocalization over five-membered ring A of the isatin moiety (Figure S13).^31^ Meanwhile, the LUMO electron cloud in IPP1 is distributed on both five-membered ring A in the isatin moiety and pyridine ring C in the pyrrolidinylpyridine moiety, indicating a stronger interaction between the two sections. However, the LUMO electron cloud in IPP2 or IPP4 is almost exclusively located in the isatin moiety. Accordingly, it can be observed that IPP1 has a red shift in the absorption peak compared to IPP2–IPP4 in their UV–vis spectra (Figure S14).^29^ Also, this may imply that isatin in IPP1 has a higher possibility to cooperate with other molecular sections and more effectively interact with the target protein if compared with those in other compounds. It has been frequently reported that the nonuniform charge distribution of the molecular surface has an important influence on its biological systems.^32^ The properties of the isomers depend on the directionality of the electron donor and acceptor. We believe that this structural diversity has a profound impact on their biological activity.

### Affinity Detection of the Isatin-pyrrolidinylpyridine Compounds with Tau Peptide by Microscale Thermophoresis (MST)

**Figure 2:**
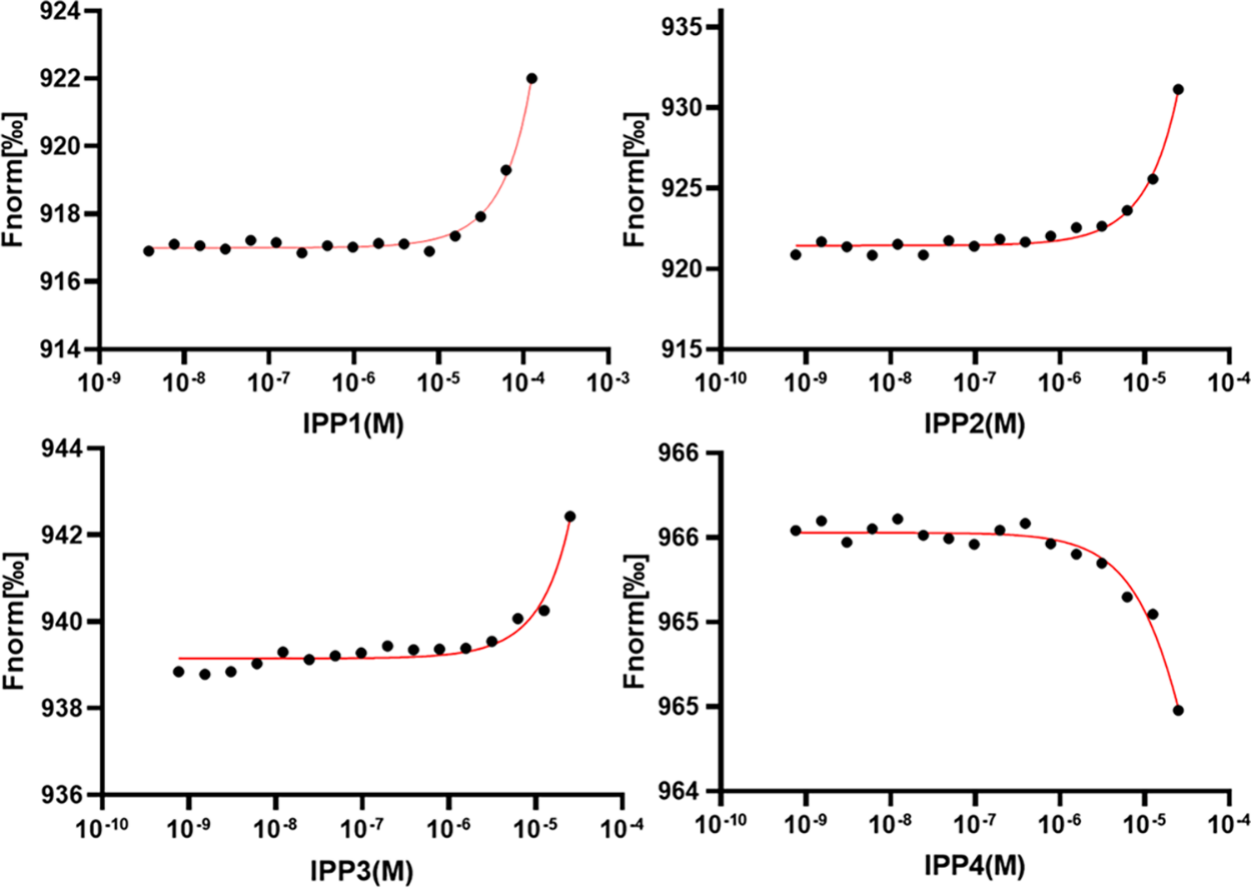
Affinity detection of IPP1–IPP4 with tau peptide by microscale thermophoresis. The affinities of these molecules with the tau peptide were analyzed by disassociation constants (*K*_d_).

Our biological exploration begins with the investigation of the interaction of the synthetic compounds with the target protein. The tau peptide R3 (^306^VQIVY KPVDL SKVTS KCGSL GNIHH KPGGG Q^336^) corresponding to the third repeat unit of the microtubule-binding domain of full tau was used as the model of a target protein because it contains the hexapeptide ^306^VQIVYK^311^, which is believed to be the nucleation site in tau aggregation.^14^ The interaction of a potential drug candidate with the target biomolecule is of great importance for the development of pharmaceuticals. The high sensitivity of thermophoresis for the binding of low-molecular-weight ligands makes MST particularly suitable to characterize the interaction of protein with small molecules in buffer. In Figure 2, the MST assay revealed the order of dissociation constants (*K*_d_) as follows: IPP1 (*K*_d_ = 20.6 ± 0.4 μM) < IPP2 (*K*_d_ = 99.5 ± 0.4 μM) < IPP3 (*K*_d_ = 266.2 ± 3.1 μM) < IPP 4 (*K*_d_ = 973.6 ± 3.8 μM). IPP1 had the highest binding affinity to tau, while IPP4 had the lowest affinity. Interestingly, we found here that these four isatin-pyrrolidinylpyridine isomers exhibit different affinities to tau peptide, although they are composed of the same functional groups with the same molecular weight. Notably, they have different molecular shapes, corresponding to the different forms of transformers. Compared to the other three isomers, the most likely reason that IPP1 binds to tau peptide with specifically high affinity is that the molecular shape of IPP1 precisely meets the protein pocket volume.^33^

### Inhibitory Efficiencies of the Synthetic Isatin-pyrrolidinylpyridine Compounds on In Vitro Tau Aggregation

**Figure 3:**
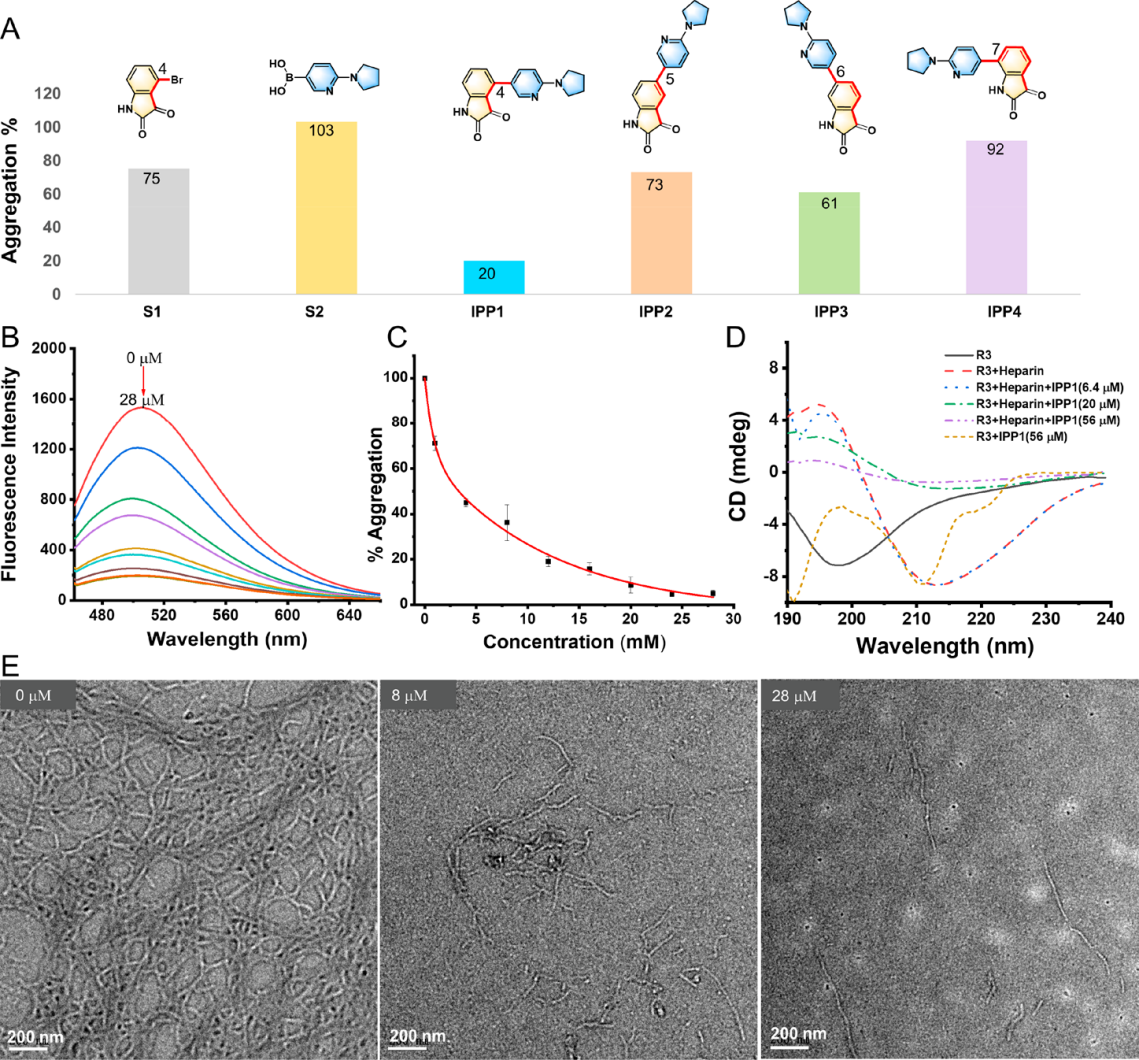
(A) Aggregation percentage of R3 (15 μM) in the presence of isatin-pyrrolidinylpyridine derivatives (IPP1–IPP4) as well as its precursors S1 and S2 (20 μM) as the aggregation inhibitors. The aggregation was initiated by heparin (3.8 μM), and the mixture was incubated for 3 h at 37 °C. (B) Decreases of ThS (10 μM) fluorescence spectra (excitation at 440 nm) for R3 aggregation systems with increasing IPP1 (0, 1, 4, 8, 12, 16, 20, 24, and 28 μM) concentrations. (C) Dose-response curves of the R3 aggregation percentage under the inhibition of IPP1, monitored by the ThS fluorescence intensities (excited at 440 nm, emitted at 500 nm) (15 μM R3, 3.8 μM heparin in 50 mM pH 7.5 Tris-HCl buffer with different amounts of IPP1, incubation at 37 °C for 4 h). (D) CD spectra of the R3 monomer, R3 aggregates, R3 monomer with inhibition compound IPP1, and R3 aggregates with different concentrations of IPP1 as aggregation inhibitor. (E) TEM images of R3 filments in the presence of IPP1 at concentrations of 0, 8, and 28 μM (scale bar = 200 nm). The filaments were obtained by the incubation of R3 (15 μM) and heparin (3.8 μM) in a 50 mM Tris-HCl buffer at 37 °C for 3 h.

Subsequently, the inhibition efficiencies of isatin-pyrrolidinylpyridine compounds on heparin-induced tau aggregation were investigated.^18^ In the research, a widely used florescence probe, thioflavin-S (ThS), was adopted by us to monitor tau aggregation because ThS binds to tau aggregates with a larger Stokes shift (excitation 440 nm, emission 500 nm), less background, and a higher detection sensitivity.^34,35^ Tau peptide R3 (15 μM) was dissolved in 50 mM Tris-HCl buffer (pH 7.4) solution, and the inhibitor, the synthetic compound (IPP1–IPP4), and the precursor (S1 and S2) were added to the reaction mixture (final concentration 20 μM) individually. Aggregation was induced by heparin (3.8 μM). After 3 h of incubation at 37 °C and the addition of ThS (15 μM) to the mixture immediately before detection, the fluorescence spectrum was recorded for 460–650 nm emission, 440 nm excitation. The aggregation percentage was calculated by decreasing the ThS fluorescence intensity (at 500 nm) of the solution with or without the inhibition compound. As shown in Figure 3A, the most significant decrease in ThS fluorescence intensity was observed in the presence of IPP1, indicating that IPP1 has the highest efficiency to suppress tau aggregation. However, IPP3 has a low inhibitory efficiency, while IPP2 or IPP4 has almost no inhibitory effect on tau aggregation. Again, this result shows that although these synthetic compounds are composed of the same molecular fragments and have the same functional groups, their inhibition effects on the aggregation of tau peptide in solution are significantly different. The change pattern has similarities with the aforementioned thermodynamic binding constants; for example, IPP1 has the largest binding constant, corresponding to the strongest inhibitory effect. However, there does not exist a strict correlation between affinity and inhibition; for example, among IPP2–IPP4, a greater binding constant does not mean a higher inhibitory efficiency. This phenomenon cannot be explained by the molecular diversity in the substituent alone according to the traditional structure-activity relationship (SAR). And other factors such as the molecular shape should be considered for a complete understanding of the SAR. Therefore, we would conduct molecular docking later to gain a deeper understanding of IPP1’s interaction with the binding site of R3.

The dynamics of R3 aggregation at different concentrations of inhibitor (IPP1) was monitored by ThS fluorescence, and a dose-response curve was then depicted accordingly, as shown in Figure 3B,C. As the concentration of the inhibitor IPP1 increases, the ThS fluorescence at 500 nm gradually decreases, indicating dose-dependent inhibitory behavior of IPP1 on R3 aggregation. At a concentration of 28 μM, IPP1 completely inhibits R3 aggregation. The half maximal inhibitory concentration (IC_50_) of IPP1 was calculated to be 3.2 μM according to the dose-response curve. Interestingly we found that IC_50_ of IPP1 was much lower than that of the inhibitors reported earlier, i.e., cyanidin (25 μM), tannic acid (92 μM) and curcumin (7 μM), in inhibiting R3 aggregation.^36−38^ The inhibitory effect of IPP1 on R3 aggregation was also confirmed by transmission electron microscopy (TEM) images, as shown in Figure 3E. It was observed that the R3 filaments appeared much less under the inhibition of 8 μM IPP1. When the inhibitor concentration reached 28 μM, R3 filaments could hardly be seen, although there sparely existed some short rod-shaped fibers.

Structural investigations of Tau, in particular at the atomic level, are hindered by its highly flexible nature. Circular dichroism (CD) is a specialized tool for determining proteins’ secondary structure and folding properties.^39,40^ In the CD experiments, we found that 30 μM was a suitable concentration at which to record the CD spectrum for R3, double that in the inhibition experiment. We kept the concentration of IPP1 between 6.4 and 56 μM, double the half-inhibition concentration, or the complete inhibition concentration, as mentioned above. As shown in Figure 3D, the CD spectrum of the R3 monomer (30 μM) was characterized by a typical random coil conformation, with a characteristic negative peak at around 198 nm. However, the CD spectrum of R3 aggregates (R3 + heparin) did not exhibit a characteristic peak typical for a single conformational structure, and a negative band at approximately 218 nm and a positive band at 195 nm probably indicated a mixture of a β-sheet or β-turn conformation, together with an α-helix conformation. With increasing concentrations of IPP1 in R3 aggregates solutions and incubated for 3 h, the CD curves gradually undergo significant changes, with less negative molar ellipticity at 218 nm and less positive molar ellipticity at 195 nm, suggesting the loss of ordered conformational structure (β-sheet or β-turn, α-helix) in aggregates. In addition, when the R3 monomer was mixed solely with IPP1 (without heparin) and incubated, the conformation of the R3 peptide partially transferred from the random coil to another type of conformation, labeled by a negative band at approximately 210 nm and a positive band at 198 nm in the CD spectrum. Although it is difficult to accurately determine which type of conformation it is, we can be certain that this conformation is different from that in the R3 aggregates. We speculate that such conformational transfer may result from the binding of IPP1. Like a molecular clip, IPP1 adheres to a certain region of the R3 peptide, preventing the peptide chain from freely curling/folding. As a result, R3 peptides will be induced to transfer from a “natively unfolded conformation” to an “aggregation noncompetent conformation”, which is believed to be pivotal to the inhibitory effect.

**Figure 4:**
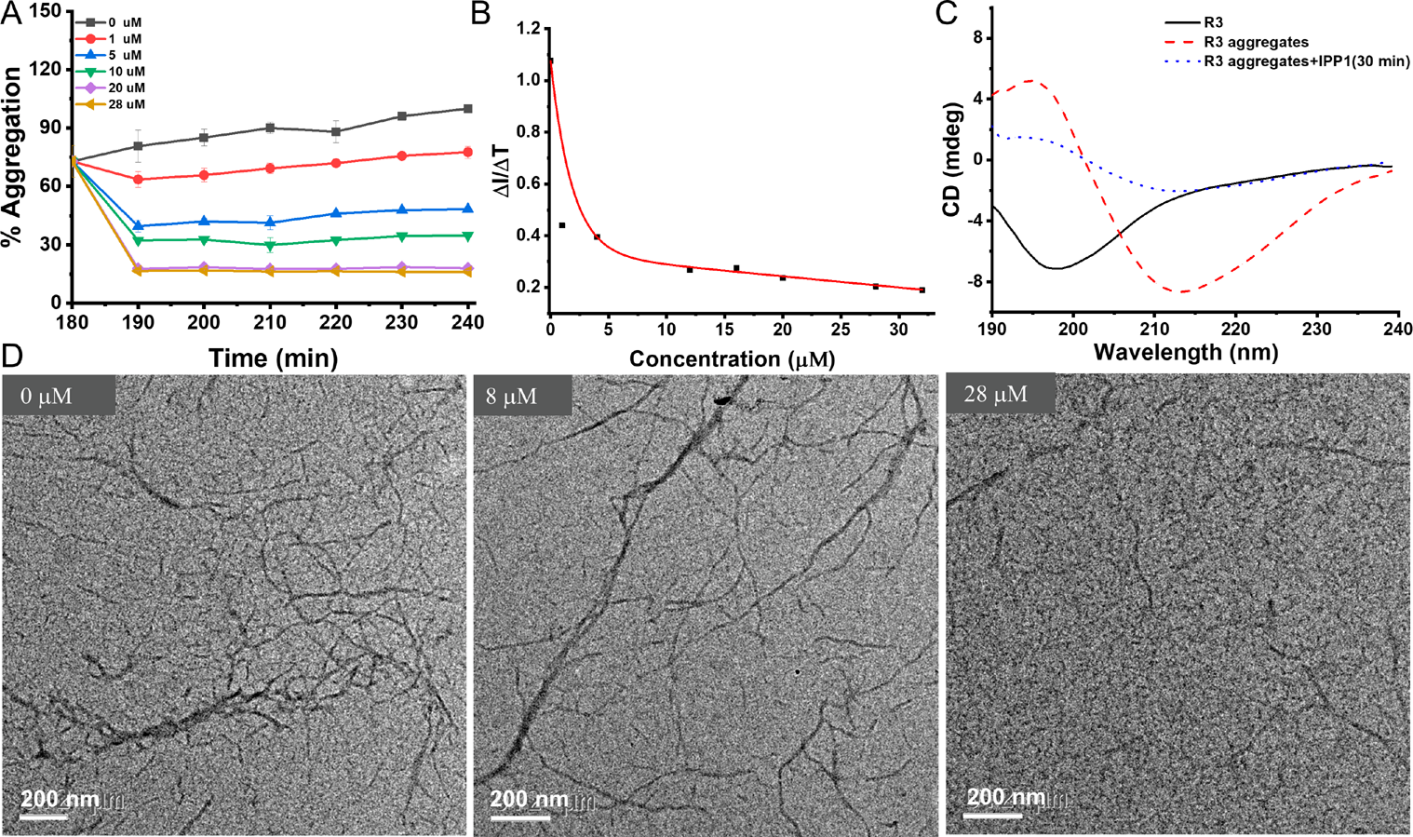
(A) IPP1 disaggregates R3 fibrils in vitro. R3 fibrils were treated with IPP1 at concentrations of 0 (black ■), 1 (red ●), 5 (blue ▲), 10 (green ▼), 20 (purple ⧫), and 28 μM (goldenrod 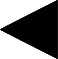). The data were reported as the mean ± SD, *n* = 3. (B) Dose-dependent disaggregate behavior of IPP1 on R3 filaments. The rate of disaggregation was illustrated by the variation in ThS fluorescence intensity within the beginning 100 s (Δ*I*/Δ*T*). (C) CD spectra of R3, R3 aggregates, and the R3 aggregate exposed to IPP1(56 μM) (0.3 h). (D) Representative TEM images of R3 filaments and the depolymerization products with 8 and 28 μM IPP1 (scale bar = 200 nm).

The superior inhibitory effect of IPP1 prompted us to further explore whether it has the ability to depolymerize R3 aggregates. Here, the R3 aggregates was obtained by incubating (3 h) R3 and heparin in Tris-HCl buffer, and the aggregation was also monitored by ThS fluorescent probe. In Figure 4A, the aggregation percentage was calculated by the decrease in ThS fluorescence intensity in the time period after IPP1 was added. It was observed that the aggregation percentage dropped significantly when IPP1 was added to the R3 aggregates’ solution, especially within the beginning 10 min. Further observation showed that the rate of depolymerization, calculated by the variation of ThS fluorescence intensity within the beginning 100 s (ΔI/Δ*T*), was concentration-dependent, as indicated in Figure 4B. The depolymerization ability of IPP1 on R3 aggregates was also confirmed by TEM imaging as illustrated in Figure 4D. It could be observed that the aggregated filaments became sparse and the length became shorter with the increased concentration of IPP1 added to the solution. The CD results (Figure 4C) also demonstrate the disappearance of the β-sheet in R3 aggregates.

### Molecular Docking and Molecular Dynamics Simulations to Predict the Possible Interaction between the Molecule Ligands and Tau Peptide

**Figure 5:**
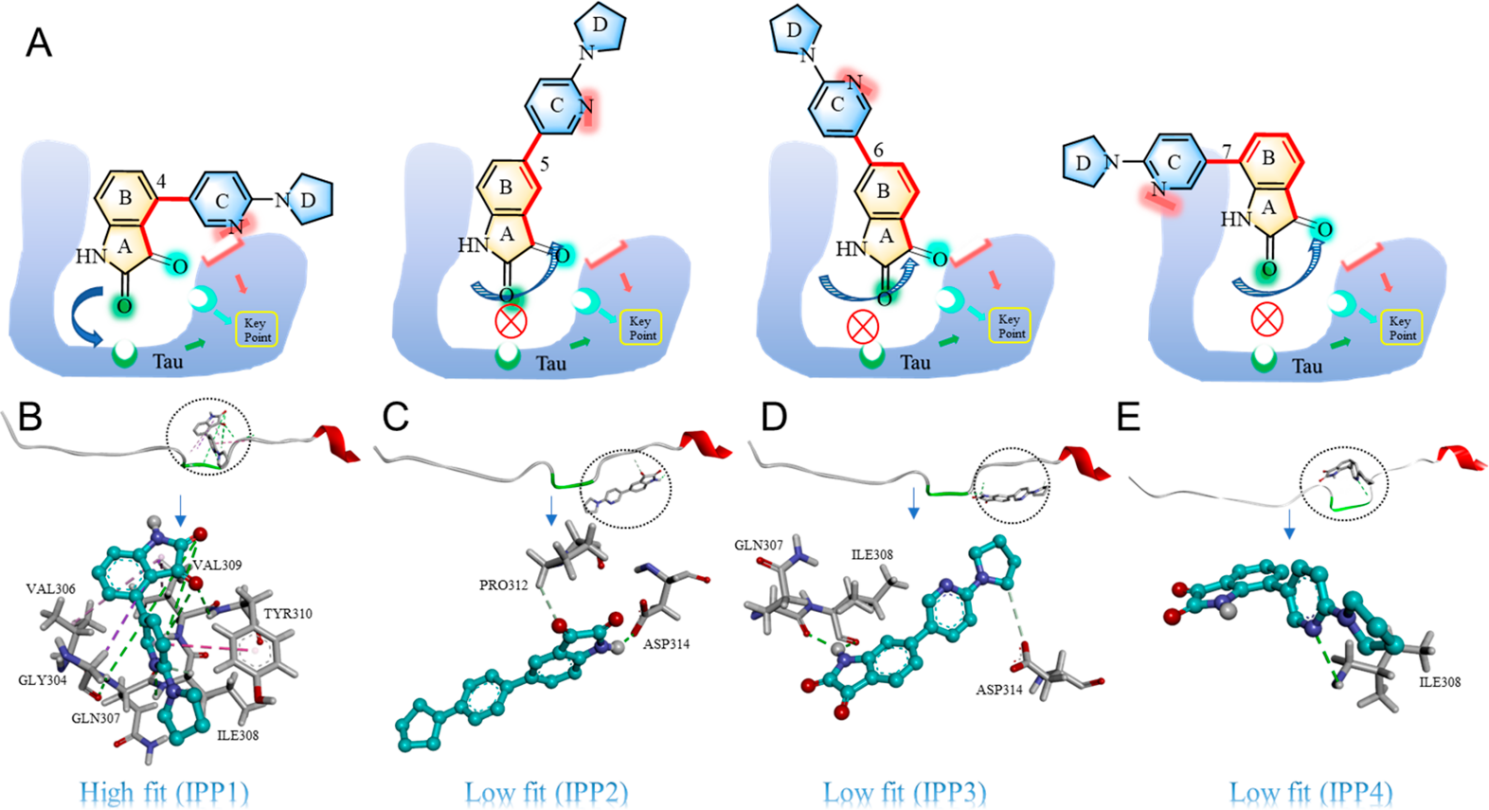
(A) General and local overview of the best-ranked docking pose of the isatin inhibitor binding with tau. The symbol (⊗) indicates that the spatial arrangement of inhibitor cannot match the active center of amino acid residues in tau peptide to form an effective interaction. Prediction of possible interaction sites among compounds (B) IPP1, (C) IPP2, (D) IPP3, and (E) IPP4 and the key fragment of the tau peptide. The carbon atoms in the compound are shown in gray, oxygens in red, and nitrogens in blue. Hydrogen bonds are shown by green dashed lines. π–π stacking or conjugation is shown by dark-red dashed lines. Hydrophobic contact is shown by purple dashed lines.

On the basis of the experimental studies described above, a molecular docking simulation was performed using Autodock 4.0^41^ in order to observe the most likely binding sites of compounds IPP1–IPP4 with the tau peptide. The crystal structure of tau peptide was obtained as a template from the Worldwide Protein Data Bank (PDB ID: 5N5B),^28^ whose amino acid sequence is 292–319 and includes the key site (^306^VQIVYK^311^). The optimized structures of IPP1–IPP4 using the B3LYP/6-31G basis set were used as the ligands. During the docking process, the IPP1–IPP4 molecules were regarded as rotatable and subjected to energy minimization. The docking result with the optimal orientation is shown in Figure 5A, and the selected docking results of the isatin-pyrrolidinylpyridine compounds interacting with tau residues are listed in Table S1.

As shown in Figure 5B, the most possible binding region of IPP1 to the polypeptide fragment is in the groove formed by ^304^GSVQIVYK^311^ (the key fragment of tau aggregation). The carbonyl group in five-membered ring A in IPP1 forms a hydrogen bond with the amino group on glutamine (GLN307), valine (VAL309), and tyrosine (TYR310). The nitrogen atom in pyridine ring C in IPP1 also forms a hydrogen bond with the amino group of isoleucine (ILE308). Pyridine ring C on IPP1 forms π–π stacking with the benzene ring on TYR310. The five-membered ring A in IPP1 also forms hydrophobic contacts with glycine (GLY304), VAL306, and VAL309, which further increases the interaction between the molecule IPP1 and tau. These results suggest that IPP1 is likely to embed in the peptide chain related to the aggregation. Visually, IPP1 is like a molecular clip that firmly holds the key region, preventing the peptide from freely curling/folding and increasing the possibility to inhibit aggregation. However, the docking results indicate that the spatial arrangement of interaction sites within the other three molecules, IPP2–IPP4, cannot perfectly match the active center of amino acid residues along the tau peptide chain. As a result, the effective interactions between these compounds and tau peptide chains are much less than those of IPP1. That means that they cannot adhere to the groove formed by ^304^GSVQIVYK^311^, as shown in Figure 5C–E, which is most likely the reason that these compounds have almost no inhibitory effect on tau aggregation. To verify the docking results, we directly applied IPP1 to the aggregation of tau hexapeptide VQIVYK (Figure S15). The fluorescence assay revealed that IPP1 significantly reduced the aggregation of tau hexapeptide induced by heparin, indicating that IPP1 as the inhibitor can directly interact with the key fragment on the tau peptide chain. The positive experimental results of IPP1 inhibition on tau hexapeptide further confirmed the reliability of our theoretical speculation on the target segment. The docking results support our assumption that the spacing fit principle is fundamental to the close recognition between drug ligands and targeted proteins, especially for intrinsically disordered proteins such as tau.

Since the molecular docking is performed on the basis of the fixed receptor conformation and accuracy-lacking scoring functions, its reliability with respect to the possibility interaction is limited, and its result can provide only collateral evidence. Therefore, we performed molecular dynamics (MD) simulations to study the dynamics between IPP and the tau peptide and obtained binding energy by molecular mechanics Poisson–Boltzmann surface area (MM-PBSA) calculations, which are more accurate than molecular docking.^42,43^

**Figure 6:**
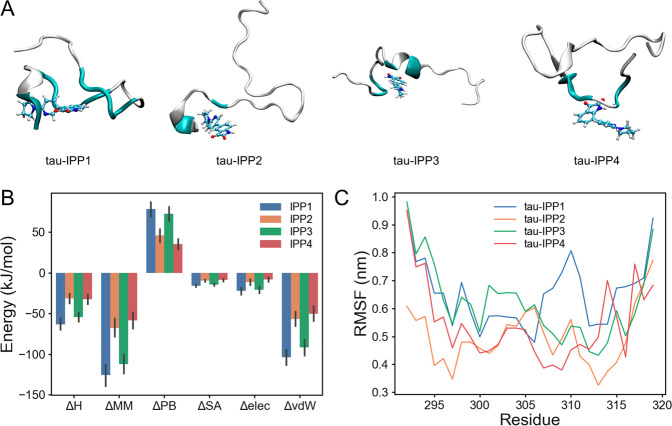
Structure of the tau-IPP complex obtained after 100 ns of MD. (A) Visualization of the conformation of compounds in complex with the tau peptide. The residues in the tau peptide that interact with IPP were colored cyan. (B) Binding energy (kJ/mol) for the interaction of tau with inhibitors using MM-PBSA analysis. (C) Comparative RMSF analysis of the tau in complex with IPP1 (blue), IPP2 (orange), IPP3 (green), and IPP4 (red).

Figure 6A shows the visualization of the conformation of IPP compounds in complex with the tau peptide calculated by MM-PBSA analysis after 100 ns in water. The binding energy (kJ/mol) analysis with MD simulations of the IPP compounds complexing with tau peptide was carried out and summarized in Table 1. Among the four IPP compounds, IPP1 has the most favorable binding affinity against tau protein with a binding energy of −63.08 kcal/mol, followed by IPP3 (−54.36 kJ/mol), and IPP4 and IPP2 had lower binding affinities (Figure 6B).

**Table 1:** Summary of the MM-PBSA Energy (kJ/mol) Component Analysis of the MD Simulation of the Synthetic Compounds Binding with Tau Peptide (PDB 5N5B)^a^

Complex	Binding energy	Molecular interaction energy	Polar solvation energy	Nonpolar solvation energy	Electrostatic energy	van der Waals energy
tau-IPP1	–63.08	–125.55	78.26	–15.79	–22.04	–103.51
tau-IPP2	–31.25	–67.58	46.02	–9.69	–11.12	–56.46
tau-IPP3	–54.36	–112.39	72.59	–14.56	–20.83	–91.56
tau-IPP4	–32.15	–58.54	35.17	–8.79	–8.17	–50.37

a Δ*H*, binding energy; ΔMM, molecular interaction energy; ΔPB, polar energy; ΔSA, nonpolar energy; Δelec, electrostatic energy; and ΔvdW, van der Waals energy.

To examine the complexation stability of the IPP compound with tau peptide, we analyzed RMSF (the Cα root-mean-square fluctuation) as a function of the residue number (Figure 6C). We also analyzed RMSD (the Cα root-mean-square deviation), the radius of gyration, and the solvent-accessible surface area of the backbone atoms of tau complexes with inhibitors over the course of 100 ns of simulation (Figure S16A–C).^44^ RMSF, which measures the fluctuations of the residues, reflects the flexibility of the residues. In Figure 6C, all of the systems have been largely stabilized after 100 ns of MD simulation. Compared to the other molecules, IPP1 caused an increase in RMSF within residue 306-211, which indirectly suggests that the IPP1 molecule has the strongest interaction with this key domain of tau aggregation. In addition, the convergence in RMSD over the simulation period provides valuable insights into the stability of the system because the fluctuation of RMSD is caused by an intrinsic property and structural instability of the tau-IPP complex rather than incomplete convergence.^45−47^ In Figure S16A,B, the IPP1 molecule showed the most significant distribution in the RMSD of the tau protein, while the IPP2 and IPP4 molecules showed smaller effects. In Figure S16C, the tau-IPP1 complex had the largest solvent-accessible surface area over the 100 ns simulation. All data are consistent with the aforementioned results. Consequently, our MD simulations further validated the results of molecular docking, which helps us to deeply understand the mechanism of inhibition effects of IPP on the tau protein.

### Investigation of the Cytotoxicity and Inhibitory Effects of Isatin-pyrrolidinylpyridine Compounds on Tau Aggregation in Human Neuroblastoma SK-N-SH Cells

**Figure 7:**
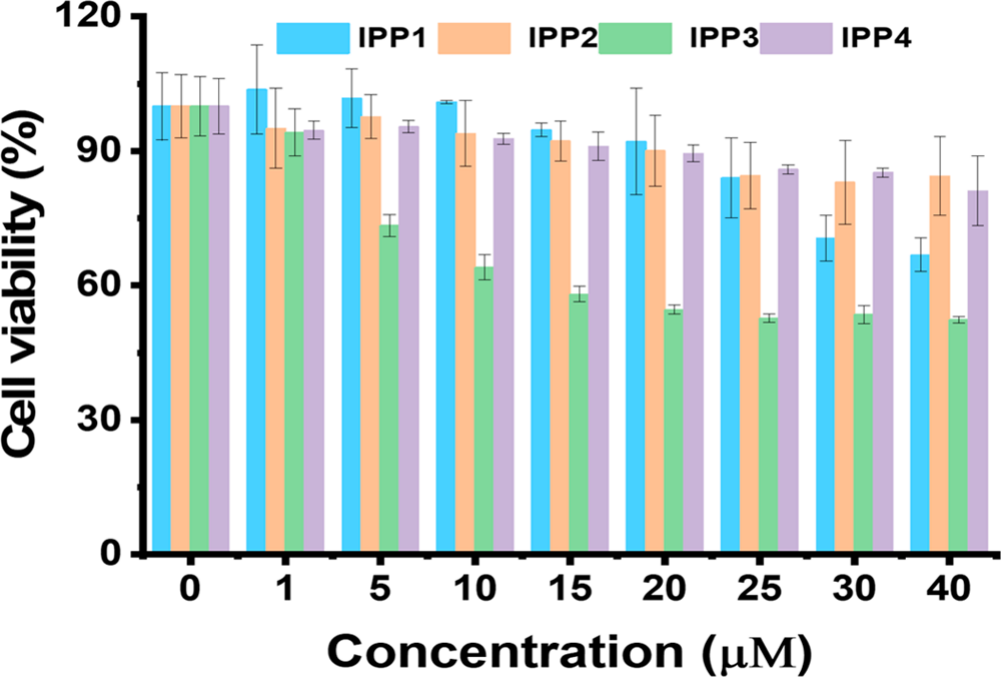
Survival rates of SK-N-SH cells exposed to IPP1–IPP4 at different concentrations, incubated for 24 h. Cell survival rates were measured with a CCK-8 cell counting kit. The data were reported as the mean ± SD, *n* = 3.

After the above-mentioned systematic study of SAR at the molecular level, we wondered whether these inhibitory effects can be reproduced in the actual biological system, laying the foundation for the discovery of drug lead compounds. Therefore, the cytotoxicity and inhibitory effects of these synthetic compounds were evaluated by human neuroblastoma SK-N-SH cells. In our previous work, we proved that tau self-aggregation in human neuroblastoma SK-N-SH cells could be induced directly by tau aggregates. The human neuroblastoma SK-N-SH cells infected by tau aggregates provide a reliable and effective cell model for the study of the tau self-aggregation mechanism.^18^Figure 7 demonstrates the survival rates of tau-infected SK-N-SH cells exposed to the synthetic compounds (IPP1–IPP4) at different concentrations for 24 h. The cell viability of SK-N-SH was assessed using a cell counting kit-8 (CCK-8) assay.^48^ It could be seen that the cell survival rate was still higher than 70% when the concentration of IPP1 reached 30 μM. Notably, although IPP2 has the lowest cytotoxicity, it has almost no inhibitory effects on tau aggregation. On the contrary, IPP3 exhibits a modest inhibitory effect as we showed previously, and its toxicity is not negligible. Consequently, IPP1 displayed superiority with relatively low cytotoxicity and a high inhibitory effect on tau aggregation in human neuroblastoma SK-N-SH cells.

**Figure 8:**
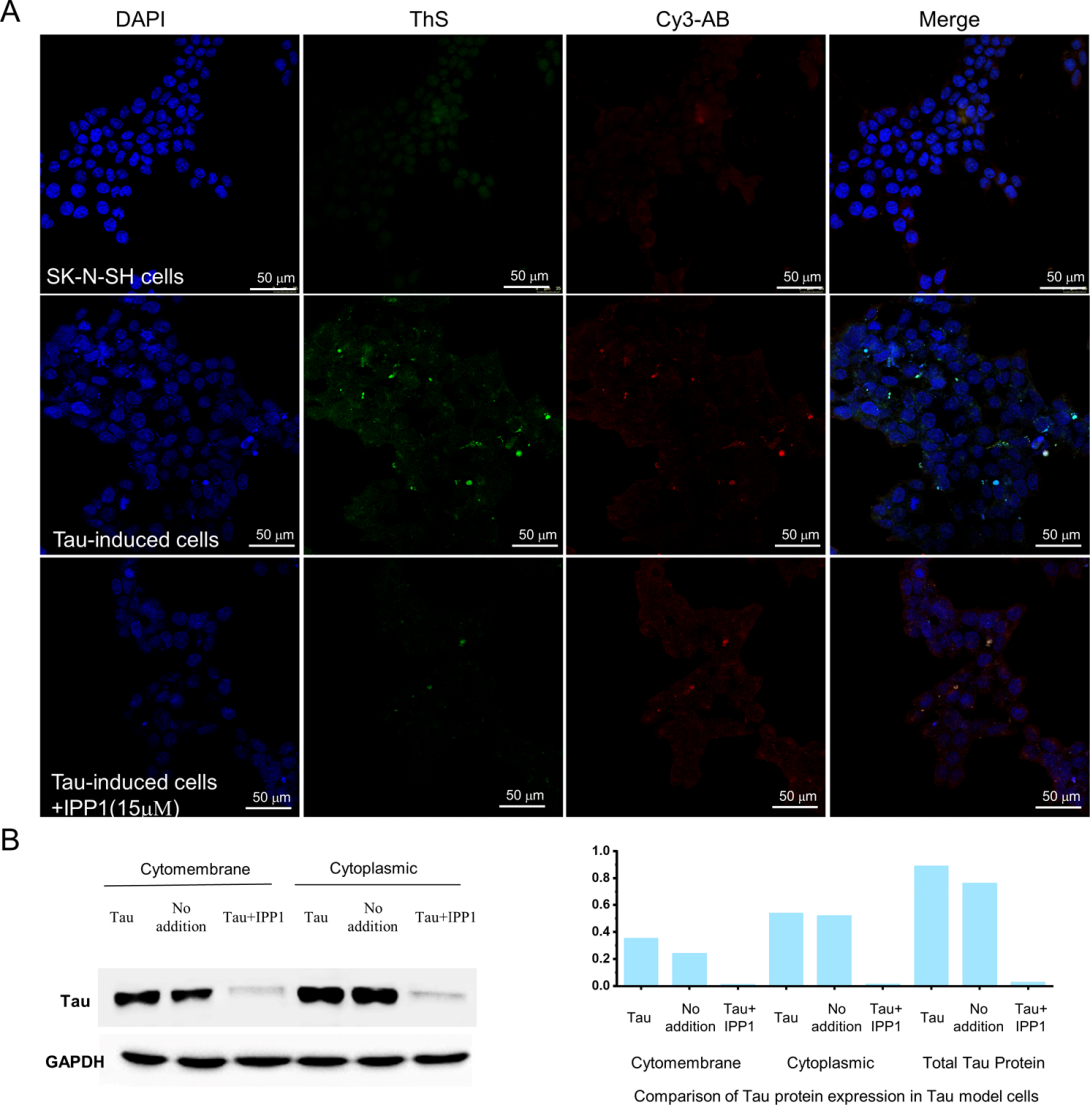
(A) Immunofluorescence staining photography. Blue fluorescence of the 4′,6-diamidino-2-phenylindole (DAPI)-labeled SK-N-SH cell nucleus; green fluorescence of the ThS-labeled aggregated tau peptide; red fluorescence of the Cy3 marked antitau antibody (Cy3-AB)-labeled pan tau protein (all conformations of tau). Scale bar = 50 μm. (B) Western blot analysis of the tau protein in SK-N-SH cells after treatment with aggregated tau fragments and inhibitors (total tau protein = tau protein in the cytomembrane + tau protein in the cytoplasm).

Using tau-infected SK-N-SH cell lines, we then conducted a series of experiments to verify the inhibitory effect of IPP1 on intracellular tau aggregation. Figure 8A shows immunofluorescence staining photographs of SK-N-SH cells (upper row), tau-infected SK-N-SH cells (middle row), and tau-infected SK-N-SH cells treated with IPP1 (15 μM) (lower row). The blue fluorescent images from DAPI staining or red fluorescent images from Cy3-AB staining show that there was almost no significant change in cell density, indicating that IPP1 did not affect SK-N-SH cell proliferation. This phenomenon was consistent with the aforementioned results of cell survival rates as measured with a CCK-8 cell counting kit. The enhanced green fluorescence of ThS and red fluorescence of Cy3-AB (middle row) indicated that tau-infected human neuroblastoma cells (SK-N-SH) were successfully induced by the tau peptide. After the tau-infected SK-N-SH cells was treated with IPP1, green fluorescence of ThS and red fluorescence of Cy3-AB (lower row) became almost invisible. However, tau aggregates in SK-N-SH cells pretreated with IPP2–IPP4 did not suffer evident changes compared to the IPP1 (Figure S17). This result demonstrates that IPP1 can significantly inhibit the aggregation of tau protein in human neuroblastoma SK-N-SH cells, consistent with the results of in vitro ThS fluorescence experiments.

Western blotting analysis of crude tau extracts from the SK-N-SH cells was also conducted in order to support the immunofluorescence staining photography result. As shown in Figure 8B, tau is mainly distributed in the cytoplasm, which is the same as reported in the previous article.^49^ After administering the inhibitor (15 μM IPP1) on the tau-infected SK-N-SH cells, only a small amount of tau was detected, a significant decrease of tau both on the cell membrane and within the cytoplasm, indicating that IPP1 had a remarkable inhibition effect on tau aggregation in human neuroblastoma SK-N-SH cells. This result agreed with the assay by immunofluorescence staining photography and aforementioned morphological observations by transmission electron microscopy.

### Primary Test of Isatin-pyrrolidinylpyridine Compound IPP1 for Clearing the Neurofibrillary Tangle on 3xTg Mice

We next sought to determine whether IPP1 could also promote tau clearance with a transgenic mouse model. Transgenic modeling of AD is a promising tool in understanding the underlying mechanisms. The triple-transgenic mouse model of AD (3xTg-AD) is the only model to exhibit both Aβ and tau pathology that is characteristic of the human form.^50^ The 3xTg-AD is an ideal model for inhibiting tau pathologies. The 3xTg AD model mice expressing APP Swedish, PSEN1M146 V, and MAPT P301L were bred in our AAALAC-accredited facility. The 3xTg mice at 10 months old were randomly allocated into a vehicle group (*n* = 3) and an IPP1 group (*n* = 3). For orthotopic brain injections and drug delivery, the mice were anesthetized with halothane (induction 5% and maintenance 1%) and fixed to the stereo tactical frame. The holes were drilled stereotaxically in the skull at a cerebroventricular location (posterior 0.22 mm, lateral 0.9 mm, and ventral 2.3 mm relative to the bregma). Using a microinjection system (Shenzhen RWD Life Technology Co., Ltd.; RWD 69100), IPP1 (5 μL, 1 mM) was diluted with 20% HP-β-CD, which was administered by direct intracranial injection, and 20% HP-β-CD solution was used as a loading control.

**Figure 9:**
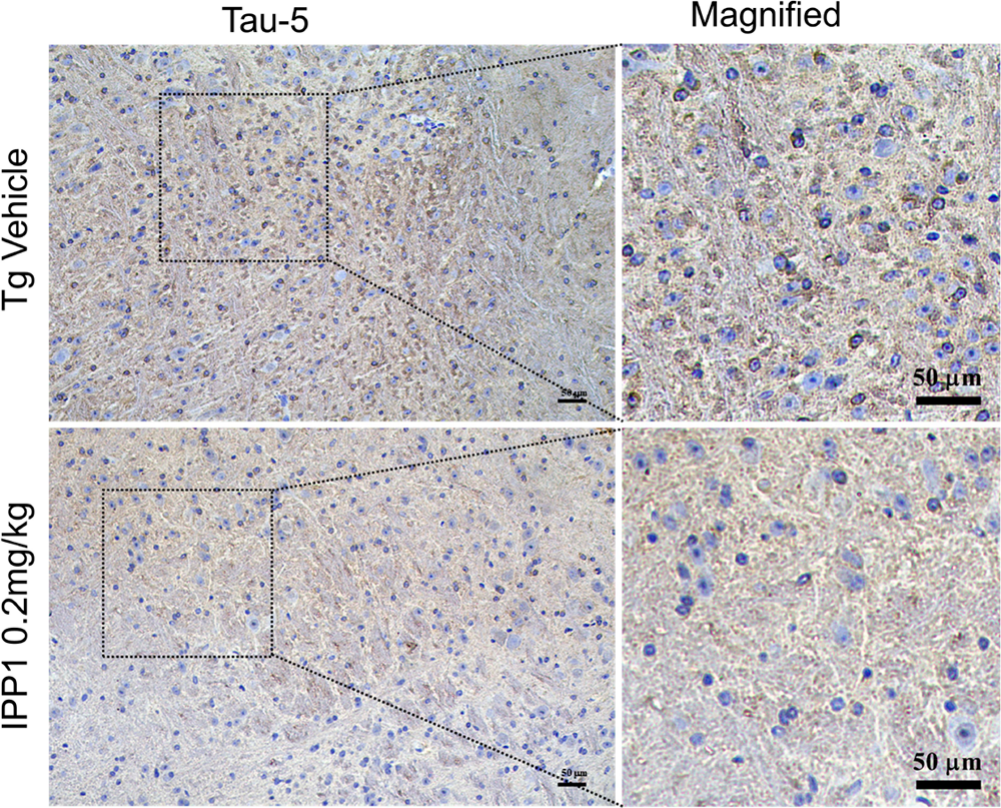
Intracerebroventricular infusion of IPP1 decreased tau in 10-m-old 3xTg AD mice. IPP1 decreased the tau level in hippocampal subsets measured by immunohistochemistry using tau monoclonal antibody (Tau-5) (scale bar: 50 μm).

It can be seen that the total tau proteins in the hippocampus were reduced following the injection of inhibitor into the ventricles as confirmed by immunohistochemistry using tau monoclonal antibody (Tau-5).^51^ The immunohistochemistry results (Figure 9) showed that the intracerebroventricular administration of IPP1 reduced total tau levels in the hippocampus. For the first time at the animal levels, the effectiveness of the direct inhibitor of the IPP1 has been confirmed. Excitingly, the above data may symbolize the possibility of IPP1 as a lead compound for future AD drug development.

## Conclusions

In this work, four new isatin-pyrrolidinylpyridine compounds were synthesized and characterized as potential inhibitors and dissociative agents for tau aggregation. The isatin-pyrrolidinylpyridine derivatives are like the different forms of molecular transformers constituted by the same functional groups jointed with different positions and orientations. MST showed that these isatin-pyrrolidinylpyridine isomers had different affinities toward tau peptide R3, the key fragment in the full tau microtubule binding domain. Among them, IPP1 had the highest binding affinity to R3 (*K*_d_ = 20.6 ± 0.4 μM) while IPP4 showed the lowest affinity. By the ThS fluorescent assay and TEM photography, it was demonstrated that these isatin-pyrrolidinylpyridine isomers had different inhibitory effects on R3 self-aggregation or even have a depolymerizing effect. Among them, compound IPP1 displayed high effective inhibitory potencies against tau aggregation. The result suggested that the introduction of a pyrazolylpyridine group at the C4 position of indoline-2,3-dione in the isatin moiety is crucial to the inhibitory potency of the compounds against tau aggregation. Theoretical calculations indicate that different molecular shapes lead to an alteration in electron distribution in molecular frontier orbitals. However, the synthetic precursor (S1, S2) and other products (IPP2–IPP4) had no significant inhibitory effect on tau aggregation. The CD experiment indicated that the transformation of tau monomers into tau aggregates is accompanied by the transformation of random fragments into β-sheets and that the β-sheet in the protein is gradually reduced or partially transformed into random fragments after the addition of the inhibitor. In combination with a molecular docking simulation, a structure–activity relationship analysis for all of these synthesized compounds was performed. It is demonstrated that in addition to the conjugated structure, substituent groups, hydrogen bond donor, etc., as previously reported, another structural characteristic that cannot be ignored is the shape of the compound for the design philosophy of the tau inhibitor. If the molecule ligand fits neatly with the peptide reception domain to maintain the correct spacing, then there exist maximum noncovalent/supramolecular interactions, namely, van der Waals forces, including the electrostatic force, hydrogen bonding, π–π stacking, and hydrophilic/hydrophobic interaction. The strongest binding should be between the molecular ligand and protein receptor. Like a “molecular clip”, the IPP1 could noncovalently bind and fix a tau polypeptide chain at the multipoint, thus preventing the transition from the “natively unfolded conformation” to the “aggregation competent conformation” before nucleation. At the cellular and animal levels, the effectiveness of the direct inhibitor of the IPP1 has been confirmed and provides an innovative design strategy as well as a lead compound for AD drug development.

## Data Availability

The data are available within the article, in the Supporting Information, or in the source data file and are available from the corresponding authors upon request. The raw NMR spectral files are available upon request. Source data are provided with this article.

## Supporting Information Available

Detailed description of the experimental procedures; materials; and additional figures as mentioned in the text (PDF)
