# Supplementary material for: Molecular Deformation Is a Key Factor in Screening Aggregation Inhibitor for Intrinsically Disordered Protein Tau
Source: ACS Cent Sci. 2024 Mar 5;10(3):717–28. doi: 10.1021/acscentsci.3c01196 (PMC10979476; doi:10.1021/acscentsci.3c01196)
Supplement: Supplementary file 1 — oc3c01196_si_001.pdf [file oc3c01196_si_001.pdf]

Supporting Information

Molecular Deformation is a Key Factor in Screening Aggregation Inhibitor for Intrinsic Disordered Protein Tau

Keke Chai[1], Jian Yang[2], Ying Tu[1], Junjie Wu[1], Kang Fang[1], Shuo Shi[1], and Tianming Yao[1*]

[1]School of Chemical Science and Engineering, Shanghai Key Laboratory of Chemical Assessment and Sustainability, Tongji University, Shanghai 200092, China;

[2]School of Medicine, Shanghai University, Shanghai 200444, China;

*E-mail: tmyao@tongji.edu.cn.

Table of Contents

Experimental Procedures 2

1. Materials and Methods 2

4-(6-(pyrrolidin-1-yl)pyridin-3-yl)indoline-2,3-dione (IPP1). 2

5-(6-(pyrrolidin-1-yl)pyridin-3-yl)indoline-2,3-dione (IPP2). 3

6-(6-(pyrrolidin-1-yl)pyridin-3-yl)indoline-2,3-dione (IPP 3). 3

7-(6-(pyrrolidin-1-yl)pyridin-3-yl)indoline-2,3-dione (IPP 4). 4

2. NMR Spectra (Figure S1~S8). 4

3. MS Spectra (Figure S9~S12). 8

4. Theoretical Studies 10

5. Molecular dynamics simulations. 10

6. IC_{50} Calculation and dynamics of Tau Aggregation and Inhibition Monitored by ThS. 11

7. Microscale Thermophoresis Measurement. 12

8. Circular dichroism (CD) spectroscopy. 12

9. Transmission electron microscopy (TEM). 12

10. Western Blot Analysis. 13

11. In Vitro Cellular Uptake and Cell Cytotoxicity. 13

12. In Vitro Evaluation of Inhibitory Effects of Inhibitor on Tau Aggregation In SK-N-SH Cell. 13

13. Animals, Treatment and Immunofluorescence. 14

Results 15

14. The contour plots of the HOMO, LUMO of all the investigated compounds (Figure S13). 15

15. UV-Visible absorption spectra of IPP1~IPP4 (Figure S14). 16

16. The selected docking results of the isatin-pyrrolidinylpyridine compounds interacting with tau residues (Table S1). 16

17. The fluorescence spectra of peptide fragments systems in the presence or absence of IPP1 (Figure S15). 17

18. Analyzing the structural stability, compactness, and solvent accessibility (Figure S16). 17

19. Representative immunofluorescence staining of tau aggregates in the three different treatment groups (Figure S17). 18

References 18

Experimental Procedures

1. Materials and Methods

All chemical reagents, including heparin sodium salt (185 USP units/mg) and thioflavin S (ThS), were purchased from J&K Scientific, TCI chemicals, Aladdin, etc. Hydroxypropyl-β-cyclodextrin (HP-β-CD) were purchased from SigmaAldrich, which used as co-solvent and antidote for IPP1 was dissolved in normal saline. Tau Monoclonal Antibody (Tau-5, anti-tau) were purchased from Thermo Scientific. All commercially available materials were procured without further purification. Reactions were analyzed by thin layer chromatography (TLC). ^{1}H and ^{13}C NMR spectra were recorded on a Bruker Advance 400/500 MHz spectrometer in $CDCl_3$ or methanol-d_6 or dimethyl sulfoxide-d_6 solutions at room temperature. Chemical shifts (δ) were quoted in ppm using the deuterated solvent resonance as an internal standard. Coupling constants (J) are quoted in hertz, and the multiplicity was defined by s (singlet), d (doublet), t (triplet), or m (multiplet). High-resolution mass spectra were measured on Bruker MicroTOF II ESI-TOF mass spectrometer.

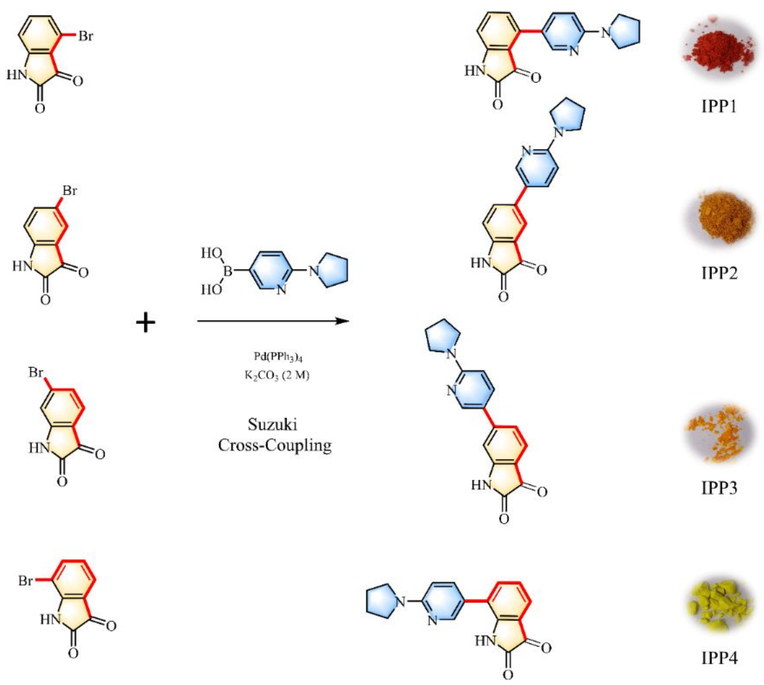

Scheme 1. Synthesis of IPP1~IPP4

4-(6-(pyrrolidin-1-yl)pyridin-3-yl)indoline-2,3-dione (IPP1).

The IPP1 was synthesized according to the previously reported method[1] with slight modifications. 4-Bromoindoline-2,3-dione(530 mg, 2.34 mmol, named S1) and (6-(pyrrolidin-1-yl)pyridin-3-yl)boronic acid (380 mg, 1.98 mmol, named S2) in anhydrous THF (50 mL) and $Pd(PPh_3)_4$ (158 mg, 0.14 mmol) was added into the round bottom flask, and then evacuated under reduced pressure, and the air was replaced with argon to fill the round-bottom flask. 50 ml K_2CO_3 (2 M) solution was added. The mixture was stirred and refluxed for 12 h at 65 °C. After TLC showed that most of the starting materials were consumed and a new main spot was formed, the reaction was cooled to room temperature. Saturated NaCl solution was added to the reaction mixture and extracted with 40 mL EtOAc three times. The combined organic layers were dried over Na_2SO_4 and, after filtering, the filtrate was concentrated under reduced pressure. The residue was purified by column chromatography on silica gel with petroleum ether and ethyl acetate to give the desired compound IPP1 as red powder, 107 mg, 18.8% yield. ^{1}H NMR (600 MHz, DMSO-d_6) δ 8.31 (s, 1H), 7.72 (d, J = 8.3 Hz, 1H), 7.63 (s, 1H), 7.55 (d, J = 7.0 Hz, 1H), 7.00 (d, J = 7.6 Hz, 1H), 6.83 (d, J = 7.4 Hz, 1H), 6.50 (d, J = 8.7 Hz, 1H), 3.45 (s, 4H), 1.97 (s, 4H). ^{13}C NMR (600 MHz, DMSO-d_6) δ: 183.5, 159.7, 157.2, 152.0, 148.7, 138.3, 138.0, 123.9, 119.6, 114.1, 110.3, 105.8, 46.9, 25.5. HRMS (ESI) Calcd. for C17H15N3O2Na $[M+Na]^+$: 316.1056; Found: 316.1057.

5-(6-(pyrrolidin-1-yl)pyridin-3-yl)indoline-2,3-dione (IPP2).

226 mg 5-Bromoindoline-2,3-dione (1.0 mmol), 192 mg (6-(pyrrolidin-1-yl)pyridin-3-yl)boronic acid (1.0 mmol), 79 mg $Pd(PPh_3)_4$ (0.07 mmol) in anhydrous THF (40 mL) and 25 ml K_2CO_3 (2 M) was added into the round bottom flask. The mixture was stirred and refluxed for 12 h at 75 °C. The other synthesis process is the same as that of IPP1. Get yellow powder 47.5 mg, 16.2% yield. ^{1}H NMR (600 MHz, Methanol-d_4) δ 8.09 (s, 1H), 7.90 (s, 1H), 7.74 – 7.68 (m, 2H), 7.47 (d, J = 10.8 Hz, 1H), 6.82 (d, J = 8.7 Hz, 1H), 6.57 (d, J = 8.9 Hz, 1H), 3.45 (s, 4H), 2.05 (s, 4H). ^{13}C NMR (600 MHz, DMSO-d_6) δ 197.90, 169.85, 155.82, 151.27, 144.56, 135.06, 132.39, 124.52, 117.81, 113.82, 107.04, 46.99, 25.45. HRMS (ESI) Calcd. for C17H15N3O2Na $[M+Na]^+$: 316.1056; Found: 316.1059.

6-(6-(pyrrolidin-1-yl)pyridin-3-yl)indoline-2,3-dione (IPP 3).

The synthesis process is the same as that of IPP2. Get yellow powder 194.3 mg, 66.2% yield. ^{1}H NMR (600 MHz, DMSO-d_6) δ 8.39 (s, 1H), 7.76 (d, J = 8.8 Hz, 1H), 7.59 (s, 1H), 7.09 (s, 1H), 6.90 (s, 1H), 6.75 (d, J = 8.3 Hz, 1H), 6.52 (d, J = 8.8 Hz, 1H), 3.42 (s, 4H), 1.96 (s, 4H). ^{13}C NMR (600 MHz, DMSO-d_6) δ 170.67, 157.06, 152.62, 146.59, 143.32, 135.50, 135.32, 122.56, 112.78, 112.31, 112.17,

106.78, 46.93, 25.46. HRMS (ESI) Calcd. for C17H14N3O2Na2 [M+2Na-H]$^+$: 338.0876; Found: 338.0871.

7-(6-(pyrrolidin-1-yl)pyridin-3-yl)indoline-2,3-dione (IPP 4).

The synthesis process is the same as that of IPP2. Get yellow powder 138.4 mg, 47.2% yield. ^{1}H NMR (600 MHz, DMSO-d_6) δ 8.05 (s, 1H), 7.61 – 7.57 (m, 1H), 7.50 (d, J = 7.8 Hz, 1H), 7.10 (d, J = 6.9 Hz, 1H), 6.74 (s, 1H), 6.60 (t, J = 7.5 Hz, 1H), 6.54 (d, J = 8.3 Hz, 1H), 3.42 (s, 4H), 1.96 (s, 4H). ^{13}C NMR (600 MHz, DMSO-d$_6$) δ 200.62, 170.45, 156.60, 149.37, 148.16, 138.06, 135.13, 133.91, 126.16, 121.33, 114.98, 114.74, 106.76, 46.89, 25.53. HRMS (ESI) Calcd. for C17H15N3O2Na [M+Na]$^+$: 316.1056; Found: 316.1059.

2. NMR Spectra (Figure S1~S8).

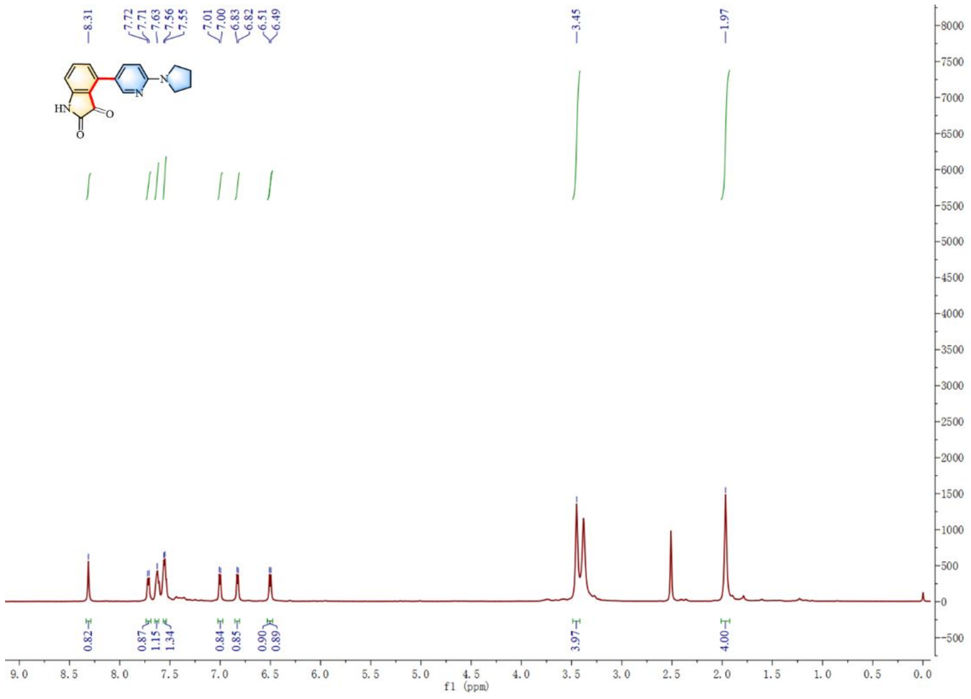

Figure S1. ^{1}H NMR spectrum of IPP1

.

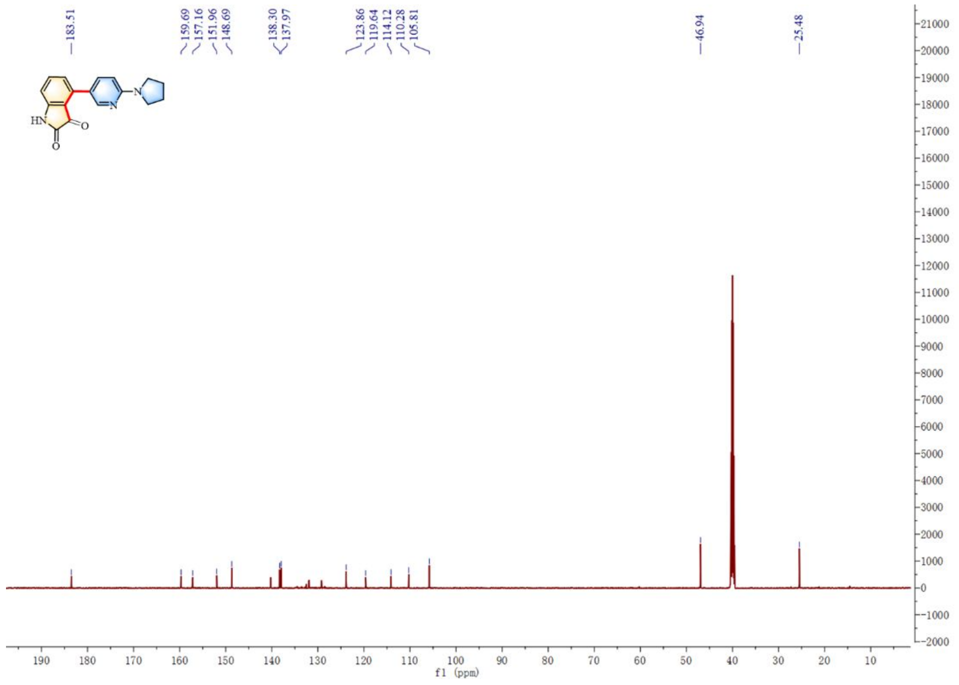

Figure S2. ^{13}C NMR spectrum of IPP1.

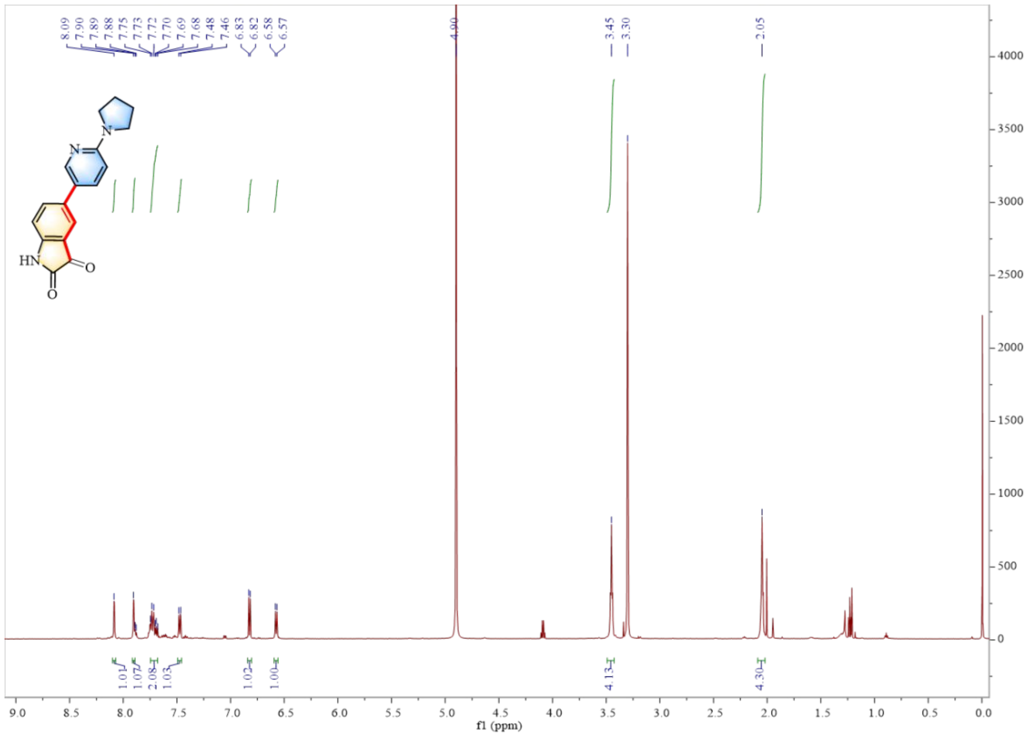

Figure S3. ^{1}H NMR spectrum of IPP2.

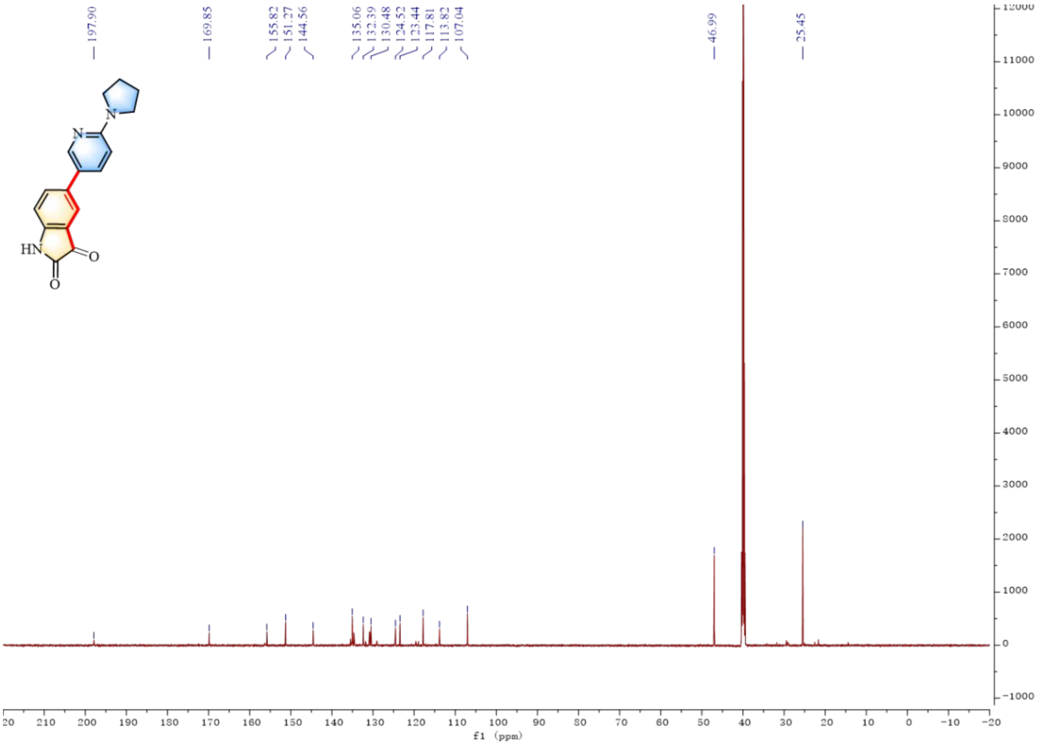

Figure S4. ^{13}C NMR spectrum of IPP2.

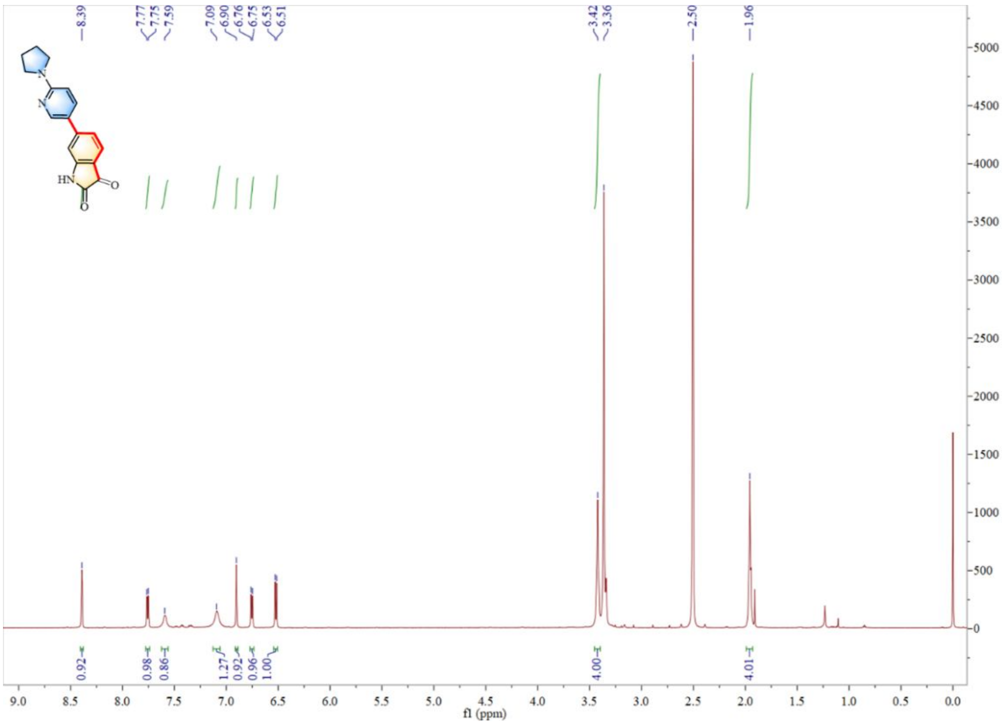

Figure S5. ^{1}H NMR spectrum of IPP3.

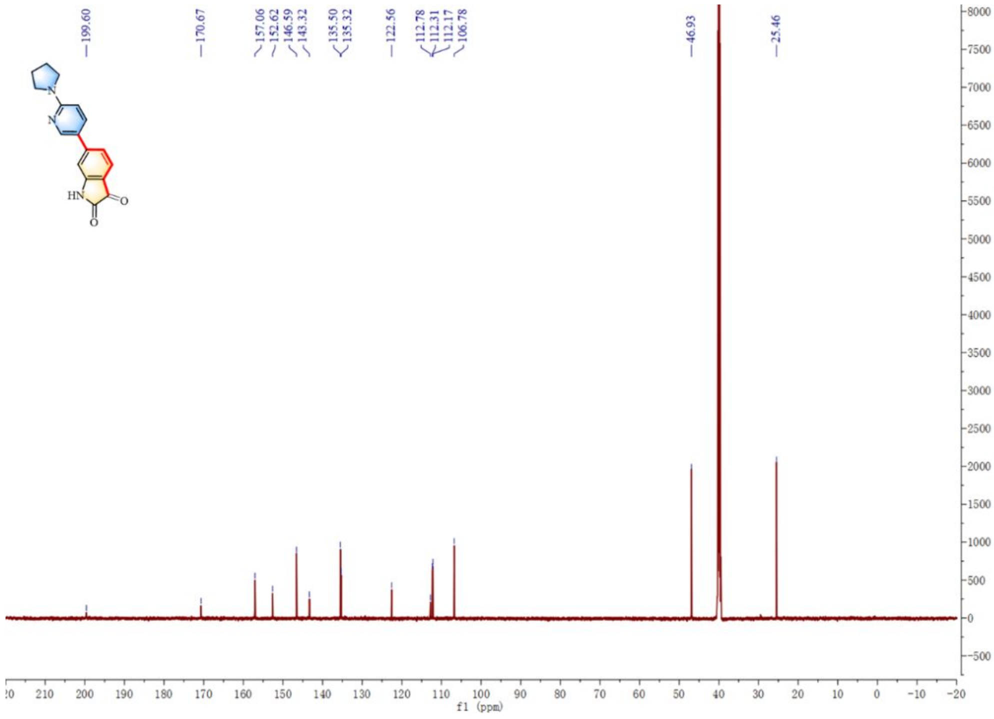

Figure S6. ^{13}C NMR spectrum of IPP3.

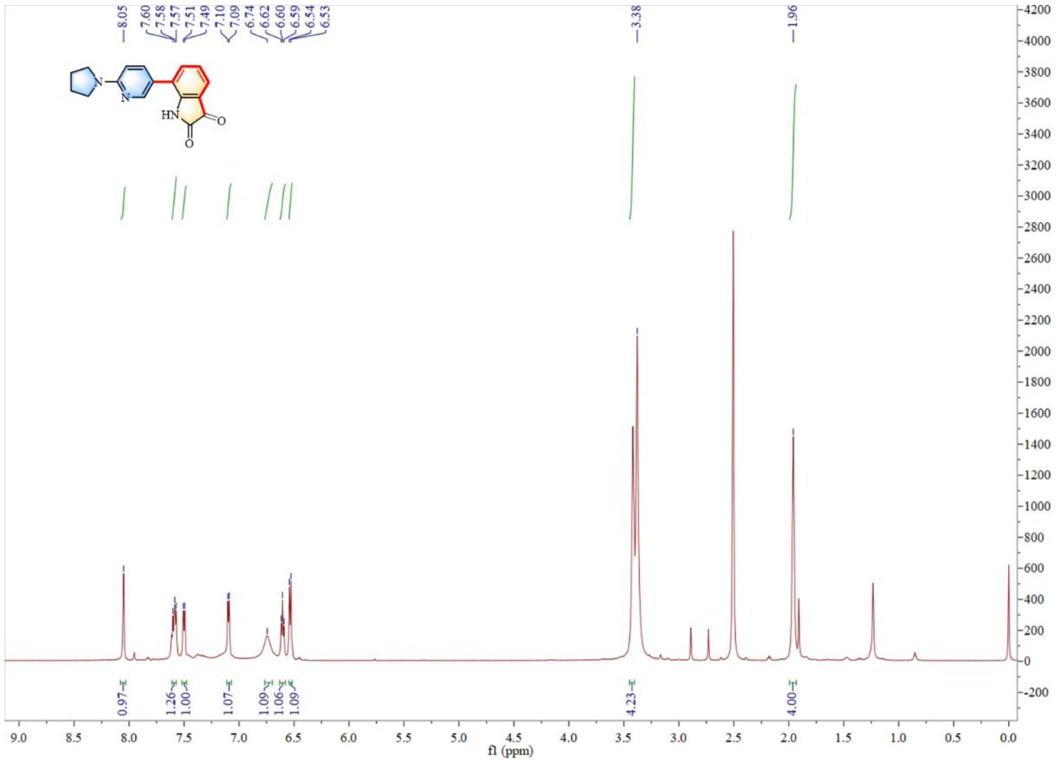

Figure S7. ^{1}H NMR spectrum of IPP4.

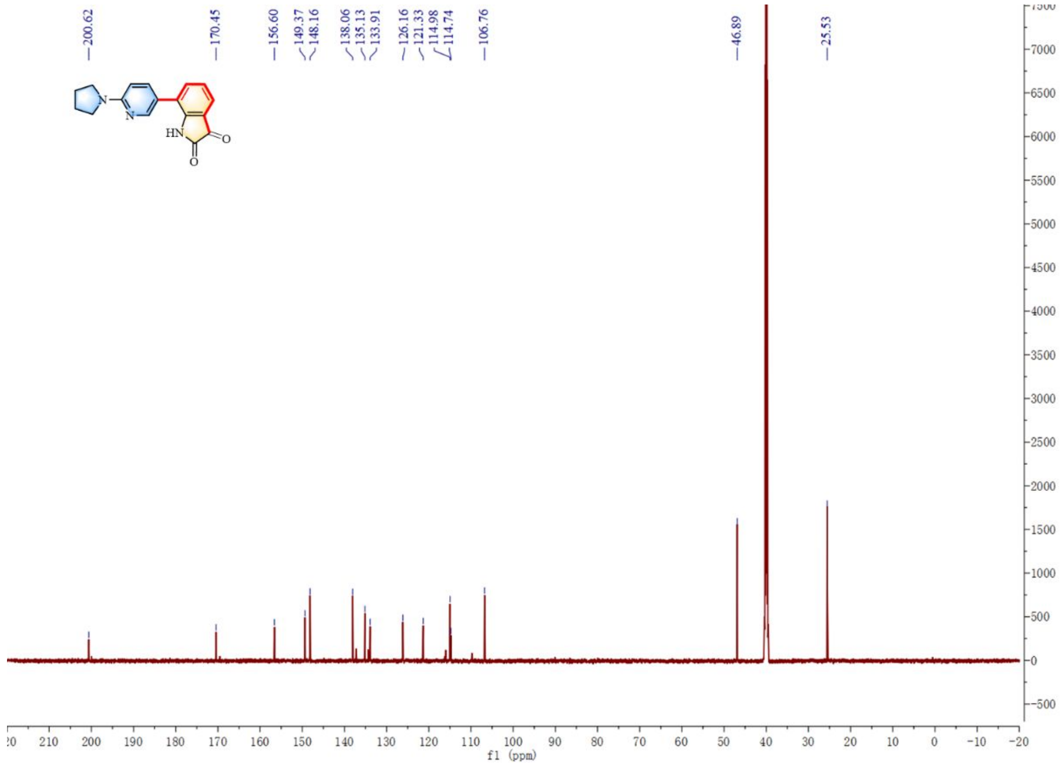

Figure S8. ^{13}C NMR spectrum of IPP4.

3. MS Spectra (Figure S9~S12).

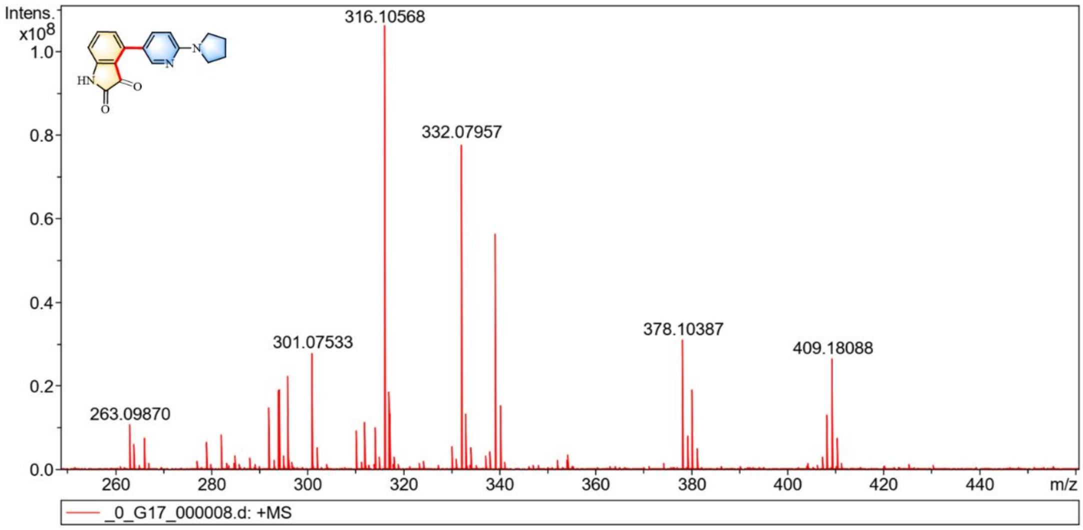

Figure S9. MS spectrum of IPP1.

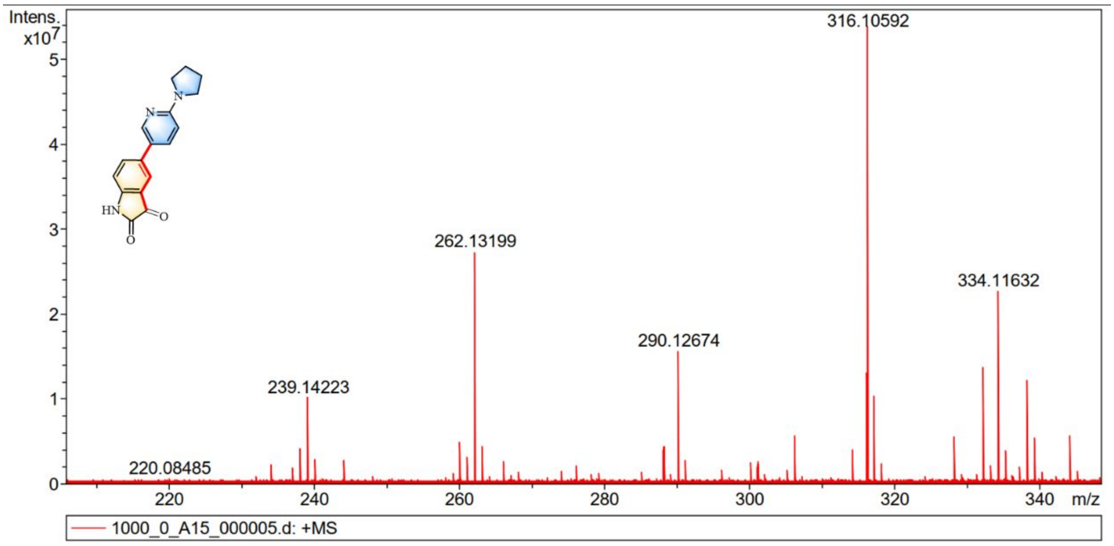

Figure S10. MS spectrum of IPP2.

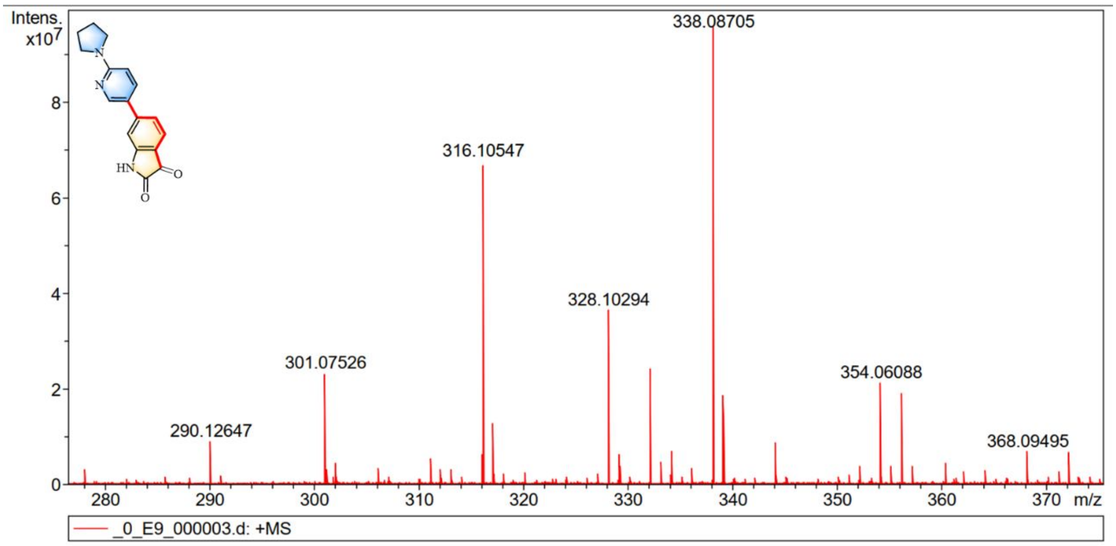

Figure S11. MS spectrum of IPP3.

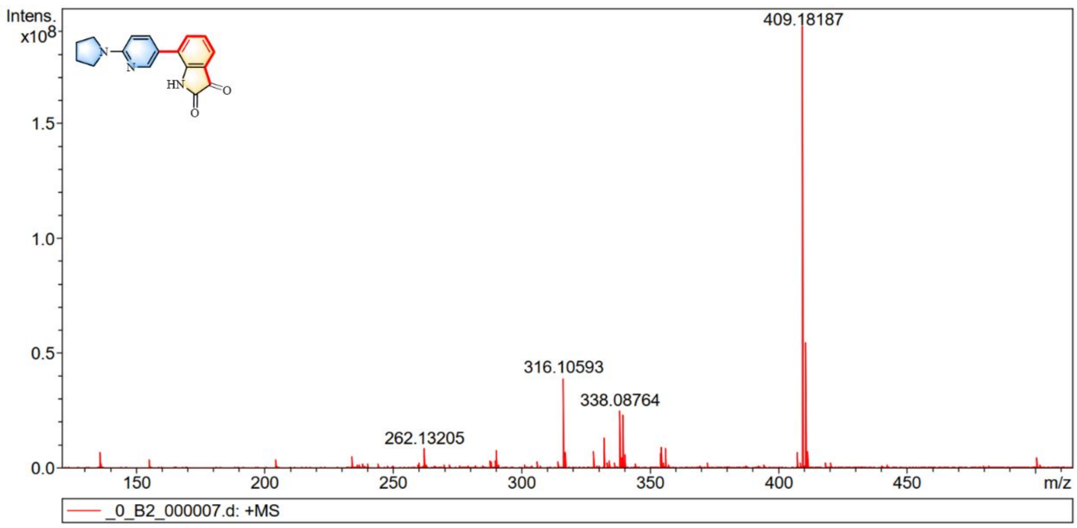

Figure S12. MS spectrum of IPP4.

4. Theoretical Studies.

Molecular docking belongs to computational chemistry, an auxiliary method for drug design based on the spatial recognition and energy recognition between molecules and the characteristics of receptors and the interaction between receptors and drug molecules. The theoretical simulation method is based on bioinformatics, which studies the interaction between molecules (such as ligands and receptors), and predicts their binding modes and affinity via a computer platform. Density functional theory (DFT) studies were applied to optimize the structures of a compound using a B3LYP/6-31G. basis set with the Gaussian 09[2]. All atoms (C, H, N, O) in small organic molecules are processed in the B3LYP/6-31G basis set[3]. The crystal structure of τ peptide was obtained as a template from Worldwide Protein Data Bank (PDB ID: 5N5B)[4], which amino acid sequence is 292 − 319 and includes the key site (^{306}VQIVYK^{311}). The interactions between tau peptide and compound were calculated by Autodock vina.4[5]. The parameter was set following previous works of our groups. Then, the results were imported into Discovery Studio version 4.5 to make further analysis and get the binding model.

5. Molecular dynamics simulations.

Molecular dynamics (MD) simulations were employed using Gromacs 2023.2 software package[6]. The Amber ff14SB force field was utilized to describe the tau protein, and the IPP small molecules were treated by acpype.py python script using the general AMBER force field (GAFF)[7, 8]. The initial states of tau-IPP complexes were taken from the binding conformations obtained from the previous molecular docking study. Each tau-IPP system was solvated with TIP3P water and neutralized with 0.15 M NaCl

to achieve electrical neutrality[9, 10]. After undergoing 2000 steps of steepest descendent energy minimization, 100 ns constant pressure and temperature (NPT) ensemble production simulations with 1 fs step length were conducted under conditions of 1 bar and 310 K, using velocity rescale thermostat and stochastics cell rescale barostat, separately[11, 12]. Finally, the trajectories were analyzed to obtain snapshots of each system at different time points. The final state conformation of tau-protein complexes was illustrated with PyMOL (www.pymol.org) and visual molecular dynamics (VMD)[13]. The contact surface area (CSA) and hydrogen bonds number between tau protein and each IPP were calculated using Gromacs utilities. The conformations among the trajectory (extracted with 1 ns time step) were clustered by *gmx cluster* command with a 0.45 nm cut-off distance using the linkage method.

The binding free energy between tau protein and IPP was calculated according to the molecular mechanics Poisson-Boltzmann surface area (MM-PBSA) method, using the super MM-PBSA program, which intrinsically calculates the molecular interaction energy including the van der Waals and electrostatic interaction energy, and obtains the solvation energy from the adaptive Poisson-Boltzmann solver (APBS) program[14], and finally achieves the ΔH and ΔG during the complex formation. Before calculation, each trajectory was treated to remove periodic boundary conditions and centered in the simulation cubic box. The interaction energy between tau protein and IPP during the whole trajectory was calculated with a time step of 1 ns. The Debye-Huckel shielding method was utilized to remove the electric screening effect. The system entropy was calculated by interaction entropy (IE) method. Finally, the binding free energy and other energy terms were exported.

6. Half Maximal Inhibitory Concentration (IC_{50}) Calculation and dynamics of Tau Aggregation and Inhibition Monitored by ThS.

Peptide tau (15 μM) is dissolved in 50 mM Tris−HCl buffer (pH 7.4) solution, and isatain pyridine derivatives as inhibitors were added to the reaction mixture. Aggregation was induced by adding 3.8 μM heparin sodium to the solution, and 10 μM ThS as a probe was added to the mixture solution, which was incubated at 37 °C for 3 h. The ThS fluorescence spectrum was recorded, respectively. The IC_{50} values of three compounds were calculated by the fluorescence intensity (excitation: 440 nm, emission: 500 nm), according to formula 1[15].

$$Aggregation\,\% = \frac{F - F_0}{F_t - F_0} \times 100$$

where F_t is the fluorescence intensity of tau induced by heparin sodium alone after incubation (tau + heparin sodium), F is the fluorescence intensity of tau induced by heparin sodium and compound after incubation (tau + heparin sodium + compound), and F_0 is the fluorescence intensity of tau alone after incubation (tau).

The kinetic test method is basically the same as the half-maximal inhibitory concentration calculation. The difference lies in the fixed measurement of the fluorescence value at the excitation wavelength of 500 nm, and the temperature kept at 37 °C by a circulating water bath. The kinetics of tau aggregation was analyzed by recording the time-dependent curve of fluorescence intensity with excitation at 440 nm and emission at 500 nm. The excitation and emission slit widths were set at 10 nm, and the voltage was 500 V. Background fluorescence of the sample was subtracted when needed.

7. Microscale Thermophoresis Measurement.

Experiments were performed using mixture of tau peptide solution and compound solution dilution series in standard grade capillaries by Monolith NT.115 instrument (NanoTemper Technologies, Munich, Germany). The tau peptide was fluorescently labeled with FITC at the 331 lysine residue and then dissolved in Tris−HCl buffer (50 mM, pH 7.5), which were incubated at 25 °C within the capillaries for 30 min prior to running measurements. Then, the protein concentration was adjusted to 0.5 μM. It was mixed with different compounds (IPP1~IPP4) respectively and analyzed by MST at a medium power and 5% LED power. The K_d values were calculated by taking the average of multiple measurements at each concentration. Data analyses were performed using MO affinity analysis software.

8. Circular dichroism (CD) spectroscopy.

CD spectra of peptide in the absence and presence of test IPP1 were measured in the 260-190 nm spectral range on a BRIGHTTIME Chirascan JASCO1500 spectropolarimeter (Jasco Corporation, Tokyo, Japan) using a quartz small cell with a path length of 1 mm and a maximum volume of 300 μL. Measurement informations were recorded at room temperature with a 1 s response, a 1 nm bandwidth, 1 nm data pitch and a 50 nm min^{-1} scanning speed. Spectra were acquired from fresh samples, which preparation method for CD Assay was same as that for the floresecence assay without thioflavin-S. All spectra were measured in phosphate buffered saline (PBS).

9. Transmission electron microscopy (TEM).

The morphology of tau filament was examined using a transmission electron microscope (JEM-2100, JEOL, Japan). Prior to TEM analysis, a mixture of 15μM tau and 3.8 μM heparin in 50mM Tris− HCl (pH 7.4) was incubated at 37 °C for 3 hours. For negative staining TEM, a copper grid was utilized. A drop of the sample solution (10 uL) was placed on the tape, followed by infiltration of the copper grid into the solution. The grid was then evaporated for five minutes and this process was repeated twice. Negative staining was carried out using 2% uranyl acetate in the same manner and subsequently dried in

a constant temperature shaker. Negative-staining electron microscopy was performed on a transmission electron microscope with an accelerating voltage of 200 kV.

10. Western Blot Analysis.

In total, 2×10^7 SK-N-SH cells/well were seeded into cell culture dishes(Φ=100mm), 24 h prior to transfection. Then, 1.5 μM IPP1 was added to Dish 3, which was incubated for 2h. After changing the culture medium, Dishes 1 and Dishes 3 were treated with 1.5 μM aggregated tau for a further 12 h at 37 °C. Then, incubation was continued for 12 h after changing the culture medium. After washing the cells with PBS, the cells were collected and lysed with RIPA lysis buffer. The cell membrane proteins and cytoplasmic proteins were extracted from cultured cells with the cell membrane protein and cytoplasmic protein extraction kit, according to the kit's instruction manual.

Proteins and buffers are mixed well for electrophoresis and poly(vinylidene difluoride) (PVDF) membranes transfer. Incubate samples overnight at 4 °C with the enclosed solution containing rabbit anti-tau antibody (Shanghai MuJin BioTech) (1:1000 of biology) and glyceraldehyde- 3-phosphate dehydrogenase (GAPDH, Shanghai Weiao Biotech 1:2000). Then, the goat antirabbit antibody and goat anti-mouse antibody (Jason 1:2000) labeled with horseradish peroxidase (HRP) were incubated at room temperature for 2 h. The membranes were reacted with chemiluminescence detection reagent (reagent A/reagent B = 1:1) for 2 minutes. Then, the films were put on the X-ray film to sensitize, develop, and fix in the darkroom.

11. In Vitro Cellular Uptake and Cell Cytotoxicity.

Cell viability was assessed using Cell Counting Kit-8 (CCK-8) assay following the manufacturer's instructions. SK-N-SH cells were seeded at a density of 2.0×10^4 cells per well in a 96-well plate and incubated for 24 h to adhere in a humidified chamber. Then, the cells were treated with various concentrations of compounds IPP1~IPP4 for 24 h. After that, the culture supernatants were removed, and 90 μL of medium and 10 μL of CCK-8 solution were then added. After further incubation for 3 h at 37 °C, the absorbance was measured at 450 nm using Microplate Reader 581 (SpectraMax iD3). Six replicates were used in each group.

12. In Vitro Evaluation of Inhibitory Effects of Inhibitor on Tau Aggregation In SK-N-SH Cell.

The effect of inhibitors on the formation of aggregate tau in SK-N-SH was tested by using the previously reported method[15]. First, SK-N-SH was grown on glass coverslips in 12-well plate at a

density of 2 × 10^5 cells per well for 24 h to adhere. Then, the previous medium was removed, for test groups, the medium containing inhibitor IPP1 or other compounds (15 μM) was added to each well and incubated for 2 h. For the control group, the culture medium without inhibitor was added. After 2 h, these cells were treated with 2.0 μM tau aggregates for 12h for another 24 hours. After that, the samples were fixed with 4% paraformaldehyde for 15 min at room temperature, then permeabilized with 1% Triton-X 100 for another 15 min at room temperature, and washed three times with PBS. Blocked with donkey serum for 30 min, incubated with rabbit anti-Tau antibody (MuJinBioTech, 1:200) at 4 ^{o}C for 24 h, and washed three times with PBS. Then they were incubated with Cy3 donkey anti-rabbit IgG (BBI Life Sciences, 1:1000) at 37 °C for 1 h, and washed three times with PBS. Subsequently, 50 μM ThS was added to stain tau aggregates for 15 min, and washed three times with PBS. Finally, cells were stained with DAPI (0.02%) for 6 min, and washed three times with PBS. The cell slides were mounted with an anti-fluorescence quencher, and the outer ring is fixed with nail polish. Fluorescence images were obtained under a confocal microscope (Leica TCS SP8).

13. Animals, Treatment and Immunofluorescence.

The 3×Tg AD model mice expressing APP Swedish, PSEN1 M146V and MAPT P301L were bred in our AAALAC-accredited facility. The 3×Tg mice (stock number, AM03201) were originally obtained from Wukong Biotechnology and transferred to C57BL/6J bedground by crossing with wild-type C57BL/6J for 12 generations. The 3×Tg mice developed AD phenotypes from 6 months old, such as tau tangle, Aβ deposits, gliosis, and cognitive deficits[16]. The 3×Tg mice at 10 months old were randomly allocated into 3×Tg group (n = 3), and IPP1 group (n = 3). All mice were kept at 24 ± 2 ºC with accessible food and water under a 12 h light/dark cycle. All animal experiments were approved by the Ethics Committee of Shanghai University (Approval Number: CSHU2021-206).

For orthotopic brain injections and drug delivery, the mice were anesthetized with halothane (induction 5% and maintenance 1%) and fixed to the stereo tactical frame. The holes were drilled stereotaxically in the skull at a cerebroventricular location (posterior 0.22 mm, lateral 0.9 mm, and ventral 2.3 mm relative to bregma). Using a microinjection system (Shenzhen RWD Life Technology Co., Ltd; RWD 69100), IPP1 (5 μL, 1 mM) was diluted with 20% HP-β-CD, which administered by direct intracranial injection, and 20% HP-β-CD solution was used as a loading control.

After 48 hours of IPP1 administration, mice were euthanized and brain tissue collected using the immunohistochemistry procedures described above. Brain sections were fixed in 4% (vol/vol) paraformaldehyde for 30 min at room temperature and permeabilized in 0.5% Triton X-100 (vol/vol) diluted in PBS solution. Free sites were blocked via incubating in 5% (wt/vol) BSA containing 0.1%

Triton X-100 (vol/vol) for 30 min. The samples were incubated with primary antibodies at 4 ^{o}C overnight, followed by washing 3 times in PBS and the samples were further stained with hoechest (1:1,000) for 10 min. Finally, samples were washed and mounted onto slides with 50% glycerin-PBS (vol/vol) solution. All the slides were imaged with a confocal microscope (Zeiss Carl LSM 780, Germany).

Results

14. The contour plots of the HOMO, LUMO of all the investigated compounds (Figure S13).

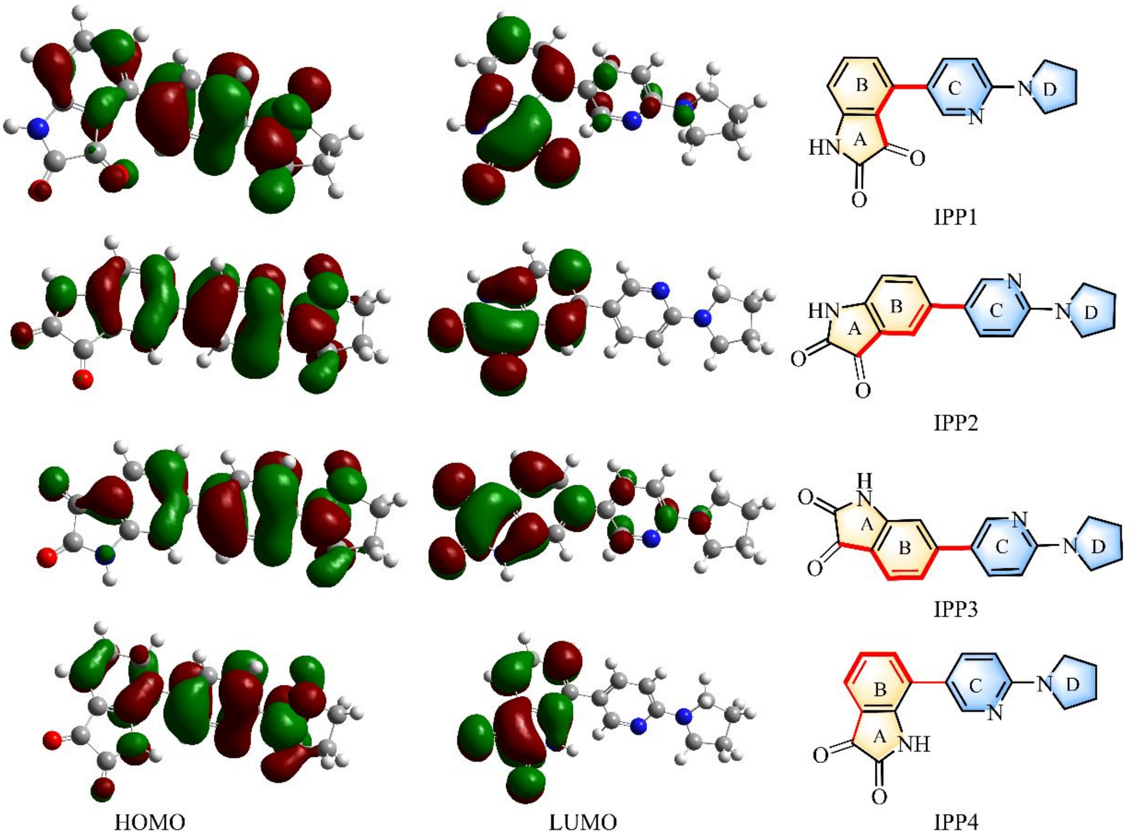

Figure S13. The contour plots of the HOMO, LUMO of all the investigated compounds.

15. UV-Visible absorption spectra of IPP1~IPP4 (Figure S14).

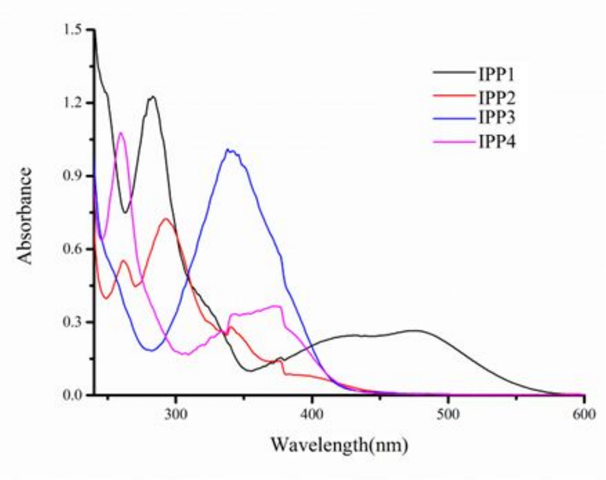

Figure S14. UV-Visible absorption spectra of IPP1~IPP4.

16. The selected docking results of the isatin-pyrrolidinylpyridine compounds interacting with tau residues (Table S1).

Table S1. The selected docking results of the isatin-pyrrolidinylpyridine compounds interacting with tau residues.

Ligand	Key residues	Interactions		
		H-Bonds	π–π stacking	Hydrophobic
IPP1	GLY304, VAL306, GLN307, ILE308, VAL309, TYR310	GLN307, ILE308, VAL309, TYR310	TYR10	GLY304, VAL306, VAL309
IPP2	PRO312, ASP314	PRO312, ASP314		
IPP3	GLN307, ILE308, ASP314	GLN307, ILE308, ASP314		
IPP4	ILE308	ILE308		

17. The fluorescence spectra of peptide fragments systems in the presence or absence of IPP1 (Figure S15).

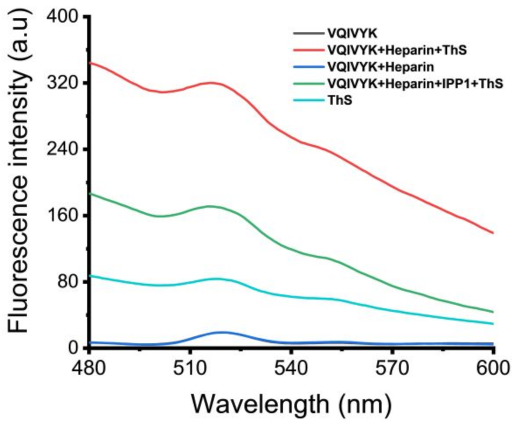

Figure S15. The fluorescence spectra of peptide fragments systems containing ^{306}VQIVYK^{311}(15 μM), ThS, VQIVYK+heparin, ^{306}VQIVYK^{311}+heparin+ThS, ^{306}VQIVYK^{311}+heparin+IPP1 and ^{306}VQIVYK^{311}+heparin+IPP1+ThS in PBS buffer solution.

18. Analyzing the structural stability, compactness, and solvent accessibility (Figure S16).

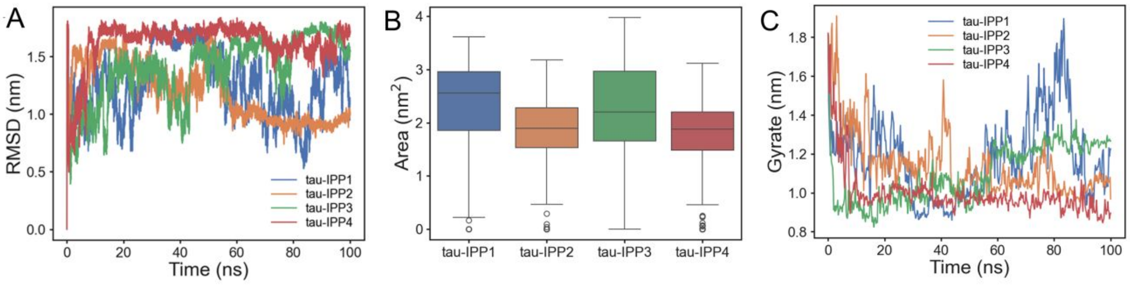

Figure S16. (A) Root mean square deviation (RMSD) of the backbone atoms of tau and inhibitors complexes over the course of 100 ns of simulation time. (B) The radius of gyrate of the backbone atoms of tau complexes with inhibitors as a function of time. (C) Solvent accessible surface area of tau complexes with inhibitors over the course of 100 ns of simulation.

19. Representative immunofluorescence staining of tau aggregates in the three different treatment groups (Figure S17).

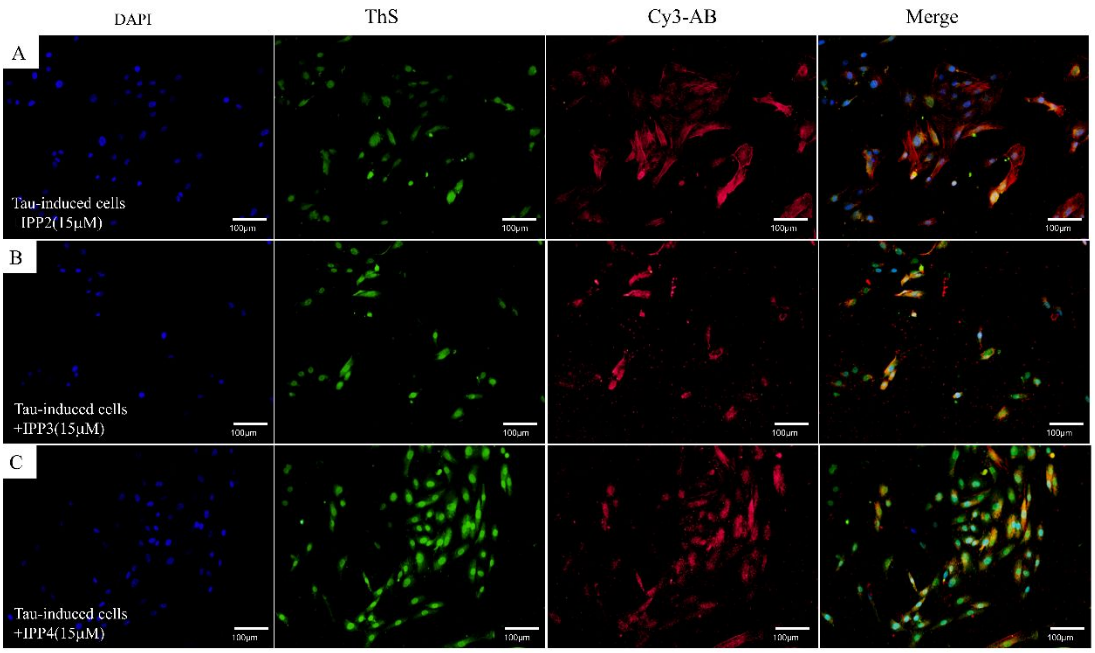

Figure S17. Representative immunofluorescence staining of tau aggregates in the three different treatment groups. (A) IPP2, (B) IPP3 and (C) IPP4. Blue fluorescence of 4′,6-diamidino-2-phenylindole (DAPI)-labeled SK-N-SH cell nucleus; green fluorescence of ThS- labeled aggregated tau peptide; red fluorescence of Cy3 marked anti-Tau antibody (Cy3-AB)-labeled Pan tau protein (all conformations of tau). Scale bar = 100 μm.

References

(1) Yang, Y.; Wang, S.; Wang, S.; Lu, L.; Zhang, Q.; Yu, P.; Fan, Y.; Zhang, F. NIR-II Chemiluminescence Molecular Sensor for In Vivo High-Contrast Inflammation Imaging. *Angew. Chem. Int. Edit.* **2020**, *59* (42), 18380-18385.

(2) M. J.Frisch, G. W. T., H. B.Schlegel, G. E.Scuseria, M. A.Robb, J. R.Cheeseman, G.Scalmani, V.Barone, B.Mennucci, G. A.Petersson, H.Nakatsuji, M.Caricato, X.Li, H. P.Hratchian, A. F.Izmaylov, J.Bloino, G.Zheng, J. L.Sonnenberg, M.Hada, M.Ehara, K.Toyota, R.Fukuda, J.Hasegawa, M.Ishida, T.Nakajima, Y.Honda, O.Kitao, H.Nakai, T.Vreven, J. A.Montgomery Jr., J. E.Peralta, F.Ogliaro, M.Bearpark, J. J.Heyd, E.Brothers, K. N.Kudin, V. N.Staroverov, T.Keith, R.Kobayashi, J.Normand, K.Raghavachari, A.Rendell, J. C.Burant, S. S.Iyengar, J.Tomasi, M.Cossi, N.Rega, J. M.Millam, M.Klene, J. E.Knox, J. B.Cross, V.Bakken, C.Adamo, J.Jaramillo, R.Gomperts, R. E.Stratmann, O.Yazyev, A. J.Austin, R.Cammi, C.Pomelli, J. W.Ochterski, R. L.Martin, K.Morokuma, V. G.Zakrzewski, G. A.Voth, P.Salvador, J. J.Dannenberg, S.Dapprich, A. D.Daniels, O.Farkas, J. B.Foresman, J. V.Ortiz, J.Cioslowski and D. J.Fox, Gaussian 09, Revision D.01, Gaussian, Inc., Wallingford, CT, 2013.

(3) Hu, X.; Yang, D.; Yao, T.; Gao, R.; Wumaier, M.; Shi, S. Regulation of multi-factors (tail/loop/link/ions) for G-quadruplex enantioselectivity of Δ- and Λ- $[Ru(bpy)_2(dppz\text{-}idzo)]^{2+}$. *Dalton Trans.* **2018**, *47* (15), 5422-5430.
(4) Cabrales Fontela, Y.; Kadavath, H.; Biernat, J.; Riedel, D.; Mandelkow, E.; Zweckstetter, M. Multivalent cross-linking of actin filaments and microtubules through the microtubule-associated protein Tau. *Nat. Commun.* **2017**, *8* (1), 1981-1981.
(5) Trott, O.; Olson, A. J. AutoDock Vina: Improving the speed and accuracy of docking with a new scoring function, efficient optimization, and multithreading. *J. Comput. Chem.* **2010**, *31* (2), 455-461.
(6) Abraham, M. J.; Murtola, T.; Schulz, R.; Páll, S.; Smith, J. C.; Hess, B.; Lindahl, E. GROMACS: High performance molecular simulations through multi-level parallelism from laptops to supercomputers. *SoftwareX* **2015**, *1-2*, 19-25.
(7) Bhadra, P.; Siu, S. W. I. Refined Empirical Force Field to Model Protein–Self-Assembled Monolayer Interactions Based on AMBER14 and GAFF. *Langmuir* **2019**, *35* (29), 9622-9633.
(8) Case. D.A.; Betz, R. M.; Cerutti, D. S.; Cheatham, T. E., III,; Darden, T. A.; Duke, R. E.; Giese, T. J.; Gohlke, H.; Goetz, A. W.; Homeyer, N.; et al. AMBER. *University of California, San Francisco* **2016**.
(9) Jorgensen, W. L.; Chandrasekhar, J.; Madura, J. D.; Impey, R. W.; Klein, M. L. Comparison of simple potential functions for simulating liquid water. *The Journal of Chemical Physics* **1983**, *79* (2), 926-935.
(10) Brotzakis, Z. F.; Lindstedt, P. R.; Taylor, R. J.; Rinauro, D. J.; Gallagher, N. C. T.; Bernardes, G. J. L.; Vendruscolo, M. A Structural Ensemble of a Tau-Microtubule Complex Reveals Regulatory Tau Phosphorylation and Acetylation Mechanisms. *ACS Cent. Sci.* **2021**, *7* (12), 1986-1995.
(11) Bussi, G.; Donadio, D.; Parrinello, M. Canonical sampling through velocity rescaling. *The Journal of Chemical Physics* **2007**, *126* (1).
(12) Bernetti, M.; Bussi, G. Pressure control using stochastic cell rescaling. *The Journal of Chemical Physics* **2020**, *153* (11).
(13) Humphrey, W.; Dalke, A.; Schulten, K. VMD: Visual molecular dynamics. *J. Mol. Graphics* **1996**, *14* (1), 33-38.
(14) Baker, N. A.; Sept, D.; Joseph, S.; Holst, M. J.; McCammon, J. A. Electrostatics of nanosystems: Application to microtubules and the ribosome. *Proc. Natl. Acad. Sci. U. S. A.* **2001**, *98* (18), 10037-10041.
(15) Hu, Y.; Yang, D.; Tu, Y.; Chai, K.; Chu, L.; Shi, S.; Yao, T. Dynamic-Inspired Perspective on the Molecular Inhibitor of Tau Aggregation by Glucose Gallates Based on Human Neurons. *ACS Chem. Neurosci.* **2021**, *12* (21), 4162-4174.
(16) Wang, W.; Zhou, Q.; Jiang, T.; Li, S.; Ye, J.; Zheng, J.; Wang, X.; Liu, Y.; Deng, M.; Ke, D.; et al. A novel small-molecule PROTAC selectively promotes tau clearance to improve cognitive functions in Alzheimer-like models. *Theranostics* **2021**, *11* (11), 5279-5295.
